# Supplementary material for: O-linked N-acetylglucosamine transferase is involved in fine regulation of flowering time in winter wheat
Source: Nat Commun. 2021 Apr 16;12:2303. doi: 10.1038/s41467-021-22564-8 (PMC8052332; doi:10.1038/s41467-021-22564-8)
Supplement: Supplementary file 1 — Supplementary Information [file 41467_2021_22564_MOESM1_ESM.pdf]

O-linked *N*-acetylglucosamine transferase is involved in fine regulation of flowering time in winter wheat

Fan *et al*.

Supplementary Method 1. Genetic backgrounds of Duster and Billings

Duster (PI 644016) and Billings (PI 656843) are two hard red winter wheat cultivars released by the Oklahoma Agricultural Experiment Station due to their wide adaptation across the southern Great Plains of the USA but with diverse genetic backgrounds [1, 2]. A doubled-haploid (DH) population of 260 lines was generated from a cross of the two cultivars, and the DH population was used to map a gene on the short arm of chromosome 1A for resistance against Hessian fly [3], *Lr34* on the short arm of chromosome 7D for resistance against leaf rust [4], as well as a yield gene on the short arm of chromosome 1B [5]. These previous studies validated the linkage groups of the DH population.

Supplementary Method 2. Positional cloning of *QHd.osu-6A*

The same set of Duster x Billings DH population was tested in a greenhouse with constant temperatures at 25/20ºC and with long days (16 h light/8 h darkness) in two experiments, one was the population without vernalization throughout the whole plant life, and the other was the population with vernalization. Vernalization was performed in a cold room with temperature at 2-6°C and the same long day as the greenhouse. The population at the 4th-5th leaf stage was moved into the cold room and returned to the same greenhouse after six weeks. Heading date was scored for each line of two populations tested with and without vernalization.

Duster and Billings were also used to generate a population of 192 F_2 plants and derived 273 F_3 plants and 153 F_4 plants that was used for screening of crossovers to happen at the *QHd.osu-6A* locus. The resulting recombinant plants were phenotyped in the same greenhouse as the DH populations were tested. Sixty plants of each critical DH line and 25 plants of each $F_{3:4}$ recombinant lines were tested under the same greenhouse conditions for fine mapping and final cloning of *TaOGT1* causing *QHd.osu-6A*.

The Duster x Billings DH lines were previously genotyped using genotyping-by-sequencing (GBS) markers, and a total of 2,358 GBS markers were identified and archived in the NCBI SRA (accession number SRP051982, https://www.ncbi.nlm.nih.gov/bioproject/PRJNA271346). A

total of 148 GBS markers of linkage group 10 was integrated with heading date from the populations tested in the field and the greenhouse to construct *QHd.osu-6A* using WinQTLCart 2.5 (North Carolina State University, Raleigh). The sequences of the GBS markers allowed a physical distance covering *QHd.osu-6A* according to the recently released IWGSC RefSeq v1.0 databases [6].

Statistical analyses on phenotypic and genetic association of five individual DH lines indicated that the target gene should be located between the two flanking markers, GBS7889 and GBS10048. Eleven internal markers were developed for fine mapping of *QHd.osu-6A*, and these internal markers allowed to identify precise locations of crossovers in five critical DH line and six $F_{3:4}$ recombinant lines. Full details of these markers are provided in Supplementary Figures 4-6.

Both Duster and Billings were identified to carry the same 2174 allele for each of *vrn-A1b*, *PPD-D1b* and *vrn-D3b* [7]. Two mutually exclusive hypotheses were designed to test if there was any new gene for heading date in the Duster x Billings population. If a QTL was associated with any of these three known genes, the QTL should reveal a new mechanism of the mapped known gene. If a QTL was not associated with any of these three known genes, the mapped gene/QTL should be new. In this study, *QHd.osu-6A* located on the short arm of chromosome 6A, in a region where was never reported to affect this trait; therefore, *QHd.osu-6A* was cloned using the positional cloning approach.

Supplementary Method 3. Quantitative RT-PCR

RNAs were extracted from leaf samples and performed using reverse transcription kit. Quantitative real time polymerase chain reaction (qRT-PCR) was conducted using the SYBR Green PCR Master Mix, and *actin* was used as an endogenous control. qRT-PCR was carried out using a 7500 Real-time PCR System (Applied Biosystems. Foster City, CA) and iQ™ SYBR® Green Supermix (Bio-Rad Laboratories. Hercules, CA), with actin used as endogenous control. There is a three-step cycling program consisting of an initial denaturation step at 95ºC for 3 min,

followed by 39 cycles at 95ºC for 15 s, 57ºC for 30 s, and 72ºC for 30 s. Primers used in qRT-PCR to amplify *TaOGT1*, *vrn1*, *vrn3*, *PPD*, *TaVRT2*, *TaGRP2*, and *actin* are listed in Supplementary Table 5.

Supplementary Method 4. MBP-*Ta*VRN1 proteins

MBP-VRN1a protein from Jagger (aa1-180) and MBP-VRN1b from 2174 (aa 1-180) used in the EMSA were from a previous study [8]. The two cDNAs were respectively cloned into pMAL-c2 vector with an MBP-tag (New England Biolabs), and were expressed in the *E. coli* (BL21). The cDNA of *TaVRT2* (DQ022679) was cloned into pMAL-c2 vector with an MBP-tag (New England Biolabs) by using the primers TaVRT2-EcoRI-F1 and TaVRT2-BamHI-R1 (Supplementary Table 5). An amylose column (New England Biolabs) was used to purify the proteins fused with MBP-tag. Purified proteins were used in the EMSA.

Supplementary Method 5. Screening and identification of interacting proteins with *Ta*OGT1

The cDNA of *TaOGT1b* was cloned in the BD vector using primers *Ta*OGT1-EcoRI-F1 and *Ta*OGT1-BamHI-R1 (Supplementary Table 5), and expressed *Ta*OGT1b was used as a bait to screen a yeast-two hybrid (Y2H) library established using whole seedlings of winter wheat cultivar 2174. Positive clone sequencing results revealed two proteins *Ta*K1 and *Ta*K4 that might have interactions with *Ta*OGT1b. The full length cDNAs of *Ta*K1 (using primers *TaK1*-Y2H-NdeI-F1 and *TaK1*-Y2H-BamHI-R1), *Ta*K4 (using primers *TaK4*-Y2H-NdeI-F1 and *TaK4*-Y2H-BamHI-R1), and *Ta*GRP2 (using primers *Ta*GRP2-Y2H-NdeI-F1 and *Ta*GRP2-Y2H-BamHI-R1) were respectively cloned into the AD vector to confirm the interactions with in co-transformation of yeast cells. The sequences of the primers are provided in Supplementary Table 5.

TaOGT1 (using primers *Ta*OGT1-BiFC-F1 and *Ta*OGT1-BiFC-R1), *TaK1* (using primers *TaK1*-BiFC-F1 and *TaK1*-BiFC-R1) and *TaK4* (using primers *TaK4*-BiFC-F1 and *TaK4*-BiFC-R1) were cloned and expressed in the pEarleyGate101 vector (pEG101) with a yellow fluorescent

protein YFP to localize the proteins. In bimolecular fluorescence complementation (BiFC) experiments, *Ta*OGT1 was fused to the C-terminal amino acid portion (aa 175–239) of YFP in the pEarleyGate202-YC vector (pEG202-YC), and *Ta*K1, *Ta*K4, or *Ta*GRP2 (using primers *Ta*GRP2-BiFC-F1 and *Ta*GRP2-BiFC-R1) was fused to the N-terminal 174 amino acid portion (aa 1–174) of YFP in the pEarleyGate201-YN vector (pEG201-YN). The primers used for cloning are provided in Supplementary Table 5. *Agrobacterium tumefaciens* strains (GV3101) carrying the BiFC constructs and p19 strain were used together to infiltrate *Nicotiana benthamiana* leaves five weeks after planting using the previous method [8].

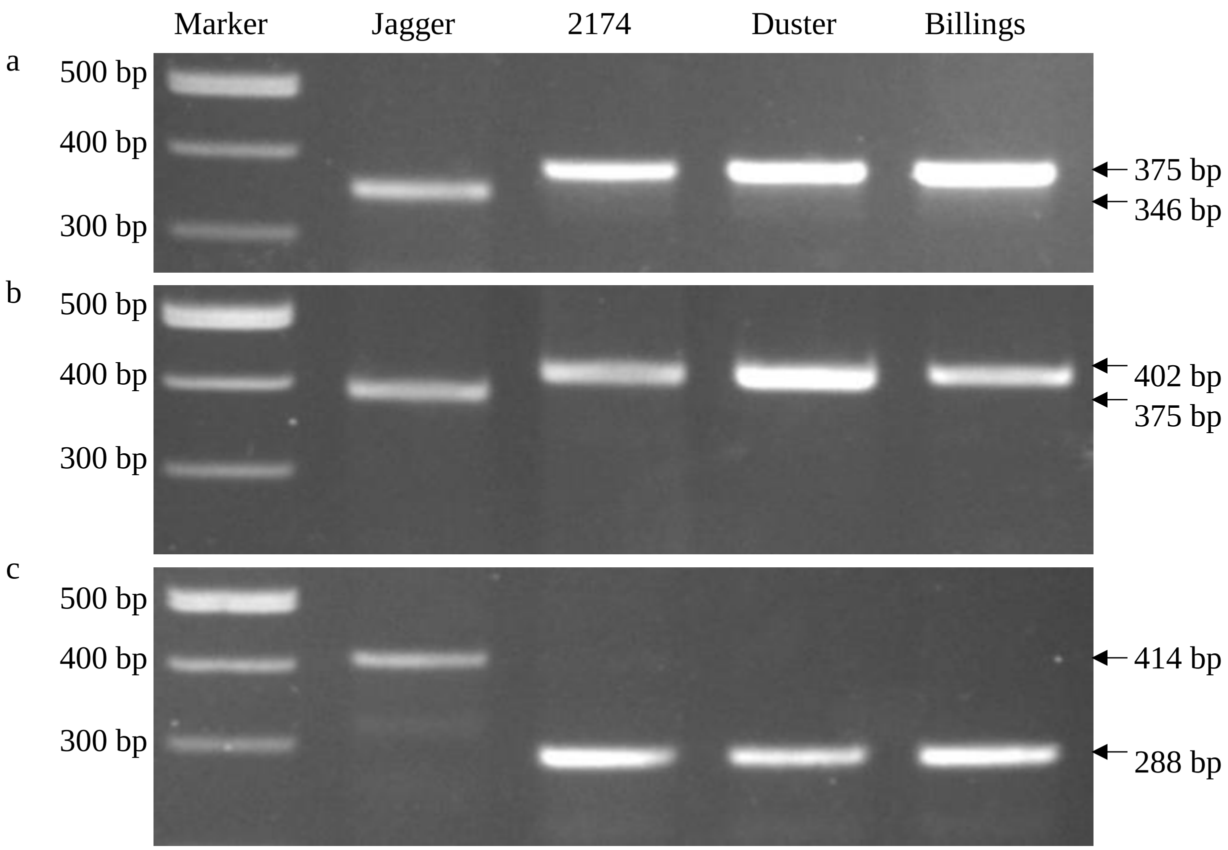

Supplementary Figure 1. Genotypes of three known genes in the parental lines. a. *vrn-A1*. Forward primer VRN-A1F4 and reverse primer VRN-A1R42 were used to amply *vrn-A*, and *PCR* products were digested with *Dpn* II. b. *vrn-D3*. Forward primer VRN-D3-F6 and reverse primer VRN-D3-R8 were used to amply *vrn-D3*, and PCR products were digested with restriction enzyme *Nco*I. c. *PPD-D1*. One forward primer PPD-D1-F and two reverse primers, PPD-D1-R1 and PPD-D1-R2, were used to amplify *PPD-D1*, and PCR products were directly checked without digestion. The digested or undigested PCR products were run on a 2% agarose gel, and distinguished DNA fragments are marked on the figures. Duster and Billings show the same alleles in these three genes, and Jagger and 2174 are used as controls. These experiments were repeated at least three times. Full gel images are provided as a Source Data file.

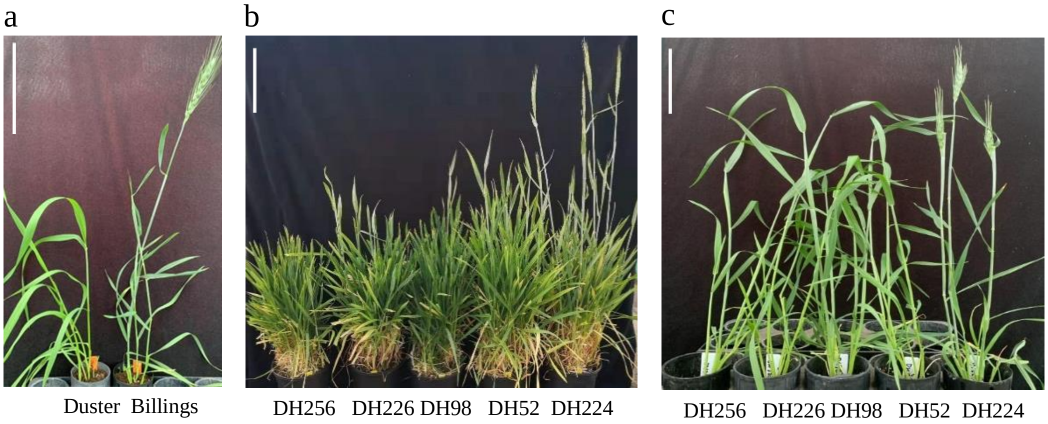

Supplementary Figure 2. Phenotypes of parental lines and critical recombinant lines. a. The two parental lines with vernalization for six weeks. b. Five critical DH lines in the same greenhouse without vernalization. c. Five critical DH lines with vernalization for six weeks. Scale bar = 15 cm.

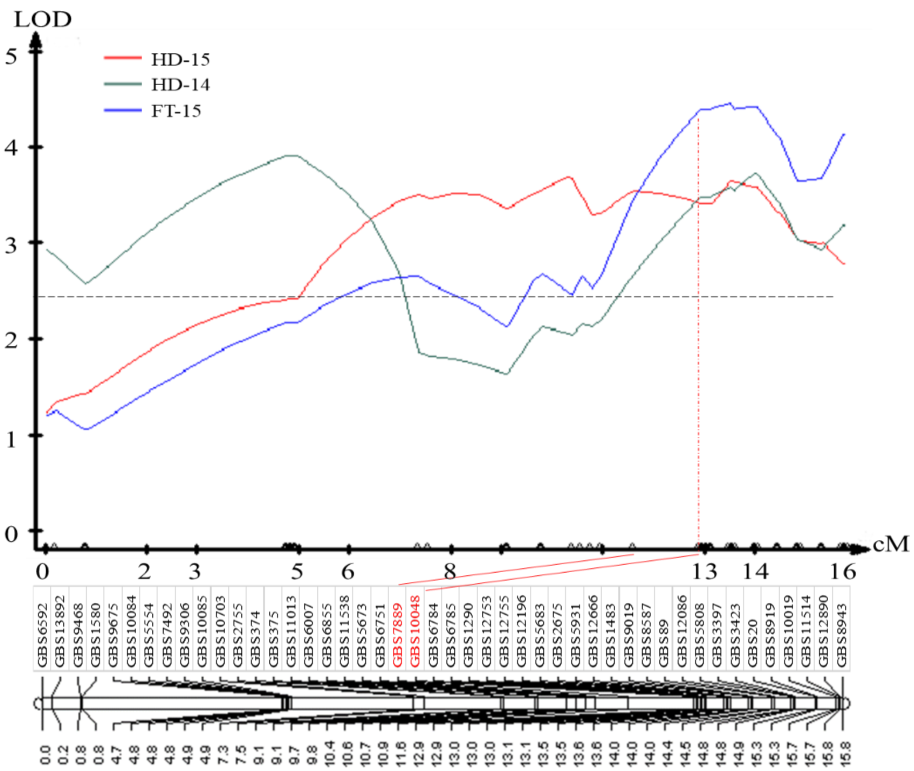

Supplementary Figure 3. QTL for heading date of the Duster x Billings DH population tested in the field. The same set of the Duster x Billings DH population was tested in the field in Stillwater Research Station, Oklahoma. In 2014, the population was phenotyped for heading date (HD-14). In 2015, the population was phenotyped for heading date (HD-15) and flowering time (FT-15). The phenotypic data was analyzed using Interval Mapping (IM) of WinQTLCart 2.5. Heading date of the Duster x Billings DH population tested in different experiments was consistently associated with the same location of *QHd.osu-6A* on chromosome 6A in wheat. Phenotype data used for QTL mapping are provided as a Source Data file.

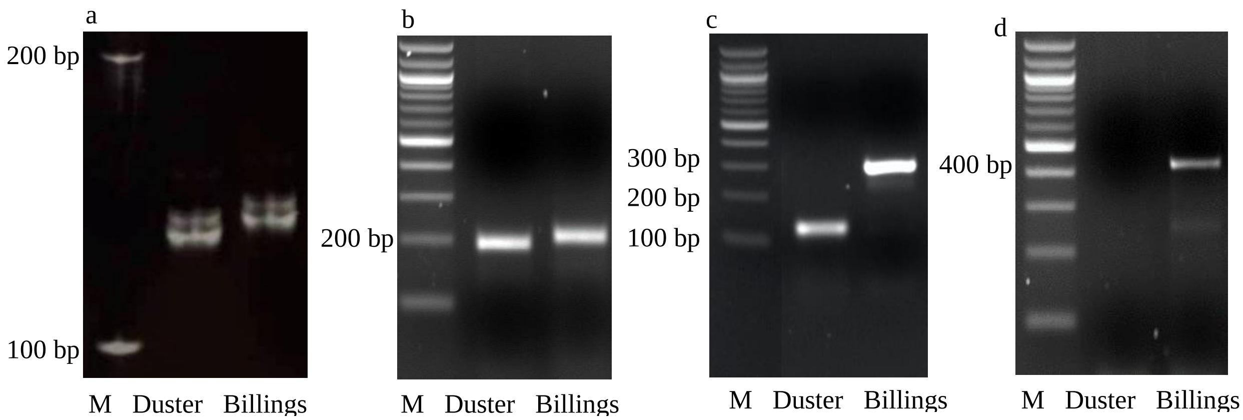

Supplementary Figure 4. Development of four PCR markers for the fine mapping of *QHd.osu-6A*. a. GTF-C3. Primers GTF-C3M-F1 and GTF-C3M-R1 were used to map variation in simple sequence repeats (SSR) in *TraesCS6A01G090600*. Duster has 2 less (CA) repeats in intron one of *TraesCS6A01G090600* compared with Billings, results in a 132 bp fragment in Duster but a 136 bp fragment in Billings. b. 6A-SSR3. Primers 6A-SSR3-F1 and 6A-SSR3-R1 were used to amplify the fragment from 60018834 bp to 60019053 bp. Billings produced the 209 bp fragment in length, whereas Duster produced a small fragment. c. *TaOGT1*. Primers TaOGT1-F1 and TaOGT1-R1 amplified a 276 bp fragment from Billings but a 108 bp fragment from Duster, due to a 168 bp indel variation (underlined) in intron of *TaOGT1*. d. 6A-SEQ9. Primer SEQ9F2 and SEQ9R2 amplified a 401 bp from Billings and a null allele from Duster. The PCRs were performed using standard protocols, PCR products were run on the 19:1 polyacrylamide gel (a, b) or a 2% agarose gel (c, d). These experiments were repeated at least three times. Source data are provided as a Source Data file.

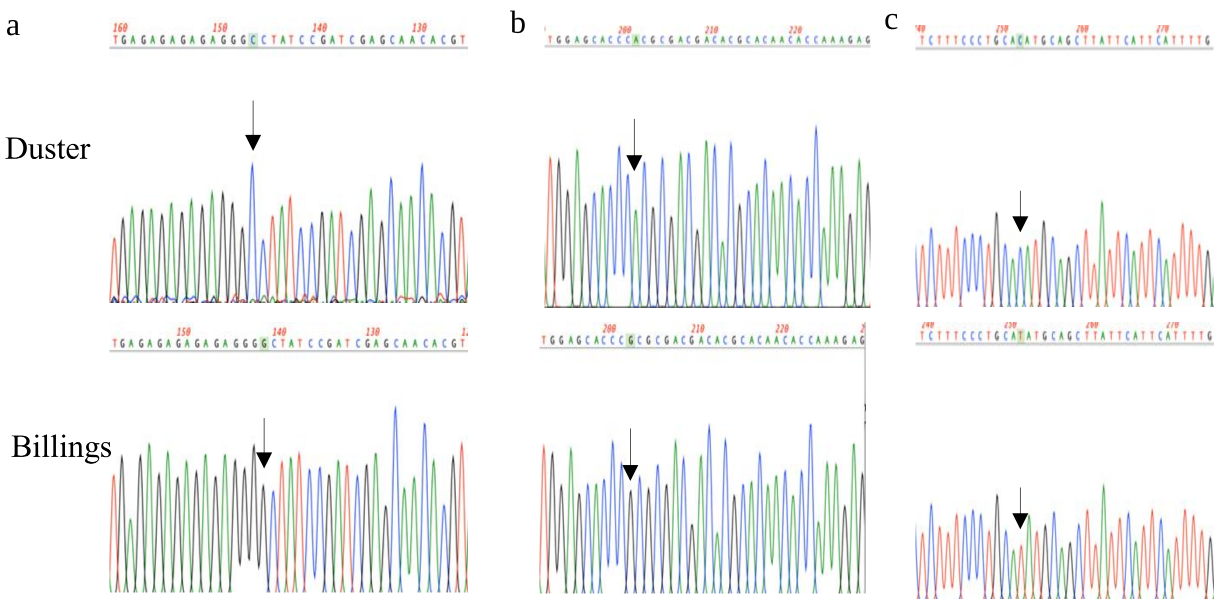

Supplementary Figure 5. Development of three sequencing markers for the fine mapping of *QHd.osu-6A*. a. 6A-400M1. b. 6A-400M2. c. 6A-SEQ7. Primers were located based on sequences by exon capture or sequences from the two alleles. The allele-specific primers (Supplementary Table 4) were designed to distinguish chromosome 6A from chromosomes 6B and 6D. PCR products were directly sequenced. The chromographic region showing allelic variation is imaged. The polymorphic nucleotide between the Duster and Billings alleles is indicated with an arrow. Sequence information of the markers is provided as a Source Data file.

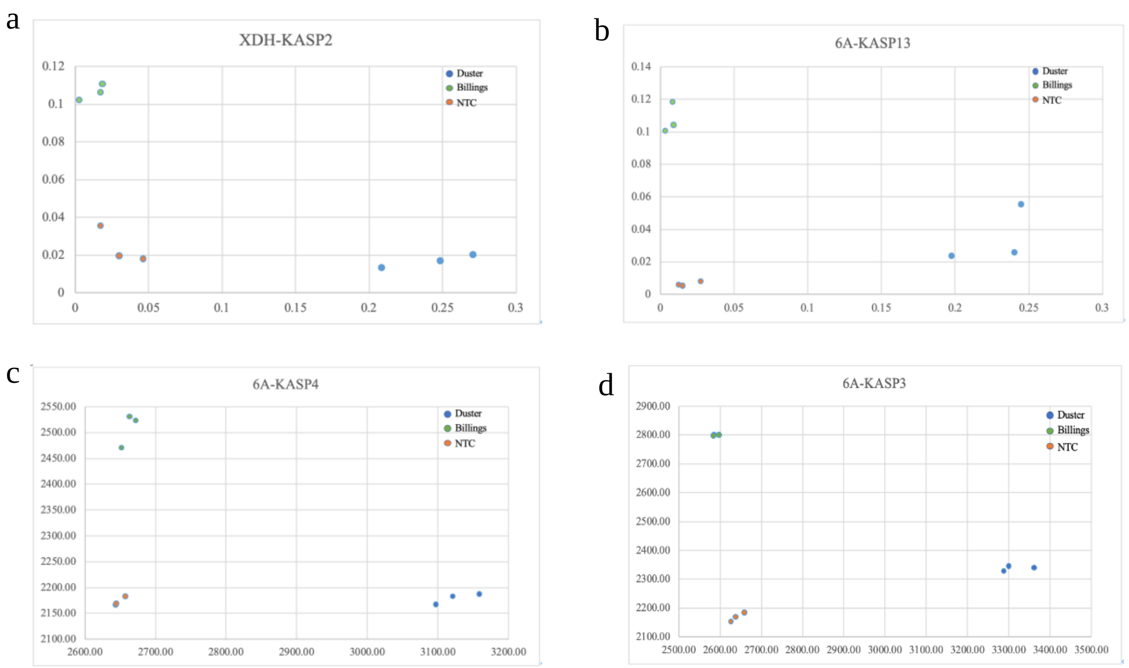

Supplementary Figure 6. Development of four KASP markers for the fine mapping of *QHd.osu-6A*. a. XDH-KASP2. b. 6A-KASP13. c. 6A-KASP4. d. 6A-KASP3. The allele-specific primers were designed (Supplementary Table 4) to carry the standard FAM (5’-GAAGGTGACCAAGTTCATGCT-3′) and HEX (5’-GAAGGTCGGAGTCAACGGATT-3′) tails. PCRs were performed in a Real-Time PCR Cycler (ABI-7500), and PCR products were read in a fluorescence scanner following manufacturer instruction. PCR cycling was performed using the following protocol: denature at 94 °C for 5 min, followed by ten cycles of touchdown PCR (94 °C for 20 s; touchdown at 61 °C initially and decreasing by −0.6 °C per cycle for 60 s), followed by 40 additional cycles (94 °C for 20 s; 55 °C for 60 s). KASP-FAM showing the Duster allele and KASP-HEX showing the Billings allele are indicated in different colors, and NTC (non-template control) used for controls is indicated in red dots. KASP genotyping data are provided as a Source Data file.

>*TraesCS6A01G091100* gDNA sequence

```
GGCTGCAGGCGTCGGAACGACGCGCCGCAGGAGCGTGGGAGCGGGAGGGAGCATAGCGAGCCGTAATATTTTTATTATTCCTTTTGAGTTCCCATCA
GGCATTTTGTATACCATGAACCATGATCCCCAAATTAATAATGTAGAAAGTAACTAGTAATTATTTGAAGATCTTTTGTGTCATTTTCTGCAGTTGA
TGCTTCCCTATAAAGCGTGTCAGTGTATGCATTACACAGCTGAAATTTGTATGCCAGTTGCGCGTCGAGTTGTGCATCCAATCAATTTGTGATGCAA
TTTCCACACCCTAAACATTTCAAATAAACTACAACTTACGAATGTATGTAGACTTATTTTAAAATGTAGATTCACTCACTTTGCTTCGTATGTAGTT
ATTTGTTGAAATCTTTAGAAAGACAAATATTTAAAAACGGAGGAAGTATAGATTCACTCACTTTGTTTTATATGTAATTACTTGTTGAAATCTCTAG
AAGTAGTAGATAGTTGATGCTTCCCTGTAATGTTTTCTCATCTGAAAAAAAAAGAGAAAACACCGGTCAGGAAATCGTTCTTTTTACAGCCAAGCCC
CCCAAAAACCTCAGCAAACCCCAAGGGACGCGGCCAAACAGGACACCCGGTCAGCCGCCCATGTTGCGCACGGCGATCGCCGCGGAGGCCCGGCGGC
GCATCTCCCTTGCCCCGGCAAGATTCGCGCACGAGCTCGCGCAGGCGCAGGCGCAGGCCCAGACGGCCGTCCCTGAGCGGGGCGGCTTTGATCCATC
GTGGGCCCCGCTCTACAGGAGAATCTCGAGGCTGTACGGCAGGCCGCCGGGAATGGTGGCGGCGGAGATGGACAACTATCTCCGCAAGCGGCGCCCG
CTCTCCGCGGACCAGATCGTCGCCTACGTCCGGAAGCTCCGCAAGTTCAAGAGCAACGCTTGCGCGCTCGAGGTGAGTCCCCACCCCCAATCTCCCC
GCAAAATTGTGGCTTGATTTGGATCGGGATGCGTTTGCGGCTTCATATATATTGCTGTTCGATGCCACGTTTGACATAGTACTAGCGTATTCGTGCT
GAAAGTAGTGGGTTCGGGATGCATCTCCATTCCGCATTCTCTTTGTGGATAATTTCGATTTAAGGCCACACTATTGGAAATGCACGATGCAAACTGA
ATGTTCCTTTTAAGACTGAAGAATGGGCCAGGGTCAACTGATTGTGTTGGCAATCACCACAATGGTAATTTGATATCTGACGCTAACTGAAACTAGG
TGCCATACTGCACATTGCTCCAGCTTCAGAGGTCAGCATTATAGGCAGGAAGGGAATTGTGTATCAGTAATAATAACGGTGATATATGTGCCGCTGC
CTGTCCTTCCTGGGAACTTTGAAGAGGTGCCGCAGCTTCCTTTTCTTTCTACCCCCTCCGTTCGGAATTACTTGTCACAAAAATAGATGTATCTACA
ACTAAAATACATCTAGATACATTCATTTCTTGGACGAGTAATTCTGAACAGAGGGAGTAAATATTATAGCTCCTTGGTTTTCACTTTTCGTTGTCTG
ATAACGTGATAGGAAATAGTATGAAAAAGATGATTGTGTATAAGATGATTTTGGCCTCAGACTTAGACACTTGGTGCATTGAGTTGAATTTTACTGA
AGTGGCTTAATAAATAAATATTAAAAGATTGGACTTAAATGACCAGTGAGTCGGTGGCTTTAGGTTCCATTACGCGTGTTGATTTAGTGCATCCATG
CAGGTGCTGACGTGTAGAGGAGGGAAGCAGATGGCTGTATTTGAGAGGGATCGACAGTCAGACCTGTTGTTATTTTAGTATAAGGGTGATGTTTTGT
AAAACAGATAGGCCACAAATGCTGGGCCTATAAGCAACATGTTGATGTACCAGCAGTTTTCTTTATGAAACCCTGGATTTTCTGTATTTGCACAAAA
ACTAGCAACTGGTGGGTGTTTGCTTAATTATAGTGTTTTTTAATTCTTGAAACAAGCTTCACTTGCTTTTGGTTGGAATTGAACAGTTTATAGATTT
TAGGAATTAGCCAGTTGTAGGTGGATACAAAGTCCTGACAAGTGAGCGGCCCTAGAGCCGGGAGGGGGCTCTGAAGGGCAATTGGAGAACTAAGGAC
ATGATAGAAGTTGGATGTTCTCCACGAACTCAGAATGTCCCGTCAGACTACAACAGCCCTCCAGTTATTTATAGCAGGGGATGTCCTAGGGTTACTT
CACATTTTACCAATATGAACTGTAAACGACGCACAGAAGTCATGTTCTCACGAATAACTGGTGGCACTATCGCCTATATGCACTTTCCATTTCTTGG
TGTCTGGTTTCAGATGTAAGATTTGGGACCTTTTGTACACCATATCTTCACCCAACGAGACCCCCTAACTCTTCCGTACATCACGTTTCTTGTTGTC
ATATCATGAGCCTCCTGTCCCTTCCCAGCGTGGCTTGGAAGTCTCTCGGCCCTGGTCCTCTGGGCGGTGGGGAGGGGGGGCATGTTGGACCGGCCCC
GGGAGATTTCTTTCGAAGCCAGGCCTTCTGGAGGCGTACGGGACATTCAAGTGGCCTTGGGTTCCGGGTACCGCCCTTTCCTCTCACAACACTGTTT
CTCGGCCTTGGGTAGGATCCTGGGAGTCCCCTCCCCTCCCTTCTTCATCCATAAGTGTCCTTTGGGCTCTCACAATGCTGTCCCTTGGTCTTGGATC
AGCCCGGGTAGGGATTGACTCGGGCGCACACTGGCACAGGAATCGGGGACCGTAATCCATTTCCCAACACCAGTAATTTAGGAATATTTGTAGAGGC
ATATGTTAAGGTTAGGACTGAATCCAAAATAATACATGTCGCTATGATAAAGAGATAGCATGGGAGCGTTTTGCTGCACTCCCATCAGAAAATGATT
CTTCTACAAACAGTATTTCCAATCAATTAGAAACTCAGTTATGAAGCTTATGATTGATAATTATGAATGCTGTCACCAGAGAGAATACATTGAGAGA
GAAAATGCATTTGCCATGAGCTAGTTCATTAGAACAGTGAATTTGTGTTGTAGTTAACGTTTCTAACAGACAATTGGTAGAAACATTTTGATGGGTA
ATTTTTTCAAACAAGCATCTCTTTCATGTGCTGTACAAATAACCGCTGATCAAACATATACAGTCTGAGATTTGAACAATGAGCTCTACCACAAAAA
CAATTGATGGTTAAGAATGGTCTTAAATAGAGTTAATTTCCAATTTTGGGGAATTAATCTGCCATTGGTAGCTTGCCCGTAACACACTCTACTGCTA
AGTCGGGGATGTCGATTCTCACACATAAATAGGCAAAAACCTGGAGAAAATATGTCCTAGAGGGAAGGAAAGTAAGACTTCACAATAGGAAATGAAG
GTTCAAGTTCTAACAGTAGAGGTTTAAATTATGATGGCATTAATGCGTCTCTCCTAAGGATCTTTTTTATGTTCACTGTAGAGATTTCATATGCTGT
TCTGGTGCAACCTCTTTCTGAGCAGAGTATCTTTTTTATGTTCACTGTATAATATCCAGCTGTGTTGTTTTGTACCCAGTTGATGGACTGGATGGAA
TCTCGTGGAGCAAAGTTGATACCTGGGCATCAAGCGCTGCGGCTTGGCCTGGTTTCAAAAGTGCATGGGATACAGGCAGCTGAAGAATATTTCTGGA
GCCTTCCTGATAAGTCAAAGAAAACTTATTCATCTTTGCTTAACTGCTATGGTGAACACAGAATGGCAAGCAAAGGTCTGGAACTCTATGAGAAGAT
GAAAGTCATGGACATTGTTCCAAATACACTGTTGTACAACAACTTGATGACTTTGTACCAAAAGGCGGGCCAGCCAGAGAAAATCCCCTCCACATTT
GAAGAAATGCGAGAAAGTGGTATAAGTGCTAACAATTTTACATATTGCATATTGATAGAAAGCTATGTTACAATGAATGACCTGGAGGCTGCAGAGA
AAGTCCTGGAAGAATTGCAGAAGGTGGCTCCAGTTCACTGGTCCTTGTACACTATGATGGCAAACAATTACCTCAAACAAGAGCTGTTTGGAAAGGC
AGAAGTGGCCCTAAAGAAAGCTGAGGAAGTCATGGATAAAGCTGAGTTGTTGTCCTGGCATTTCCTCATCTCTTTGTATGCCCGTTCTGGCAATTCA
ACCGAGCTTAAGAGGATCTGGGAGTCTTTGAAGTCCACATTCAAGAAATGCTCGAACAAAAACTATCTTGTGATGCTTGAAGCTCTGAGTATGATTG
ATGACCTTGAGTCTTTGCAGCAAATCTTTCAGGAGTGGGAATCGAGTAATGAACATTACGACATGAGGATAACAAATGTCATGATCAAAGCTTACCT
TGATAAGGGCATGATAGACGAAGCTGAGGCTATCCGTCAGAGCACCATGTCTCAGGGCCATTGCAACGGGAGGACGGTTTACATGTTTGCCGAGTTC
TATTTGGACAAATCTGATGTTACCGCGGCACTGGAGATCTTGAGAGATGCAAAGAAGATGCTTACGGCACACAAATGGGTGCCATCGGAAAAGCTTA
CGAGCAGATTTCTGAAGCACTATGAAGAGTCAAAGGATGTTGATGGTGTGGAGTCTTTCTGCGAGTGCCTGAAGAAGCTCGAATGCCTTGATGCGGA
AGCTTATGAGGGCATGGTGCGGACCTATATAGCTGCAGGTAGAACCAACCCATCCATTGCTCAGCGCATCGAAGATGATGGGATTCACGTTGGCCCT
GAGACGACGAAATTGCTGGAGCGTGTTTCCGGCAATTGAAACTGAGAAGGAAAGAAACTTTGTAAGAACTCGAGGCCACTGTAGAATTTTTCTTTAT
TATAGAGCAGAATTATGACTTGTTGAAGATGTCGTTATCTCGTGTAAAACTTGATGTCACCGGATGAAGTACATGAATTGCAAGGTCTTTTGGCATT
TTACATTTGACCCCCTGACTACTGTGTACCATCTATCTAAGCTGCTAATACGATGGAGGAGTCATTTTCAATTATTTTCATGTTGCTCAGGTACTTA
ACCAGTCGATTGCAGTACCCA
```

Supplementary Figure 7. Genomic DNA sequences of *TraesCS6A01G091100*. Primers used to isolate this gene are highlighted in green. The start codon, stop codon, GT and AG of each intron are shown in red letters and with highlighted in yellow. No SNP was observed in the sequenced region between Duster and Billings.

>*TraesCS6A01G091200* gDNA sequence

```
ACAACCATCGGGGACAGTCGGCGGAGGAGGCGTCGCGTGCGACGTGGGAGAGTGTTCTCCCCTGGGCTGGGCCAGCACCGGTGTTCATCGACCTTAC
CGCCCCGGGGAAGAGGACGACTACGACGCCGCCTAGGGGCAGTGCTGACCATGCACGTTTTTTAAAGATTTATTAACATTTAAATGAACTTTGGCC
CGCGGTTGATCGTCCATTTAATACTCAGTTATGTTAAATTATGTTCATTTTGGCCAAATGTTTTGAAACTTTTTTTGGACACGAGAAAAATGAATCT
GCCATCATCGACCCAAACAAAAAAGAGGACGCGCCAGTCCGCTCGGGTGACCCAATTAAGAGCATCTCCAATAGACGGTCCAAATGTAAAAATACTT
AACTTTTGGACCGTCTGGGGCAAAAAACCCTGCTCCAACAGACGGTCTATATGCAAAAAAAAATGGCCCGCGGCCTTCTCGTGATGTAAAATACAAC
ACTTCGCGATGCAAATATATATCACGAGATGCATCTGGTCCAAAACTAGCCGCCGCCCGCGAACGCCCAACCGCCACTTCATTTCCTTTCCCACCCG
CCCGCGCCCGTCCGCCCGCTTGCGACCCTCCCGGCCGCCGCCGTCCAGCGCCGGCATGGCCGATGTCGACGACCCCGCCGCCTTCTCCGCCGCCGCC
ACAGCCGATGGGCTGACCCACGTGGCCGCCGCCGCTACCATCCCGCCGTAACTGCGGCCATGCCGTCGCCCCCCGCCCGCCGGCCCGAATTGGCGCC
GCCCCGGCCGGTGGCCACCGCGCAGGCGGCGAAGCCGGTGAAGGGACGAGGAGGCAGTGTCAAACGGCCGGCCAACCGCAGGCGCAACCCGGCCGAC
ATTTCTACGCGGCCGGTGAAGGTCTCAAAGGCGGCCGCGGCGGTTCGAGGCAACGATTCGCAGACGGTGCGGCCGGCGGCCTCTTCCAGCAGCCACA
CATCCATTCCTCCACCTCCCCCGCCGCCACCGCCGGCCGTGGAGGAAGATGTGGCCACGCCGACGGCCACCGCCTCCAACATGTTCGACGAAATGTC
GCAAAGGTATAATTTTCGTGGTTGTGCTCTCGTTTGTTGTTTCAAATTTTTTGTGTTCTTGATTGTTTGTGCGTGCAATTATAATGTTGATGATGAA
GCTTCCATGCACACGCAAAAAATGTGGCAAATGATGATGCACTTTATTTTCAAATTTTAGTTGCTATTTCAATACAAAACCGTGGCTGAACAATGGT
TGGTAGCAGCCGCGCGGCCGTTTTCATCTTTATTTTGGATTATCTATTGAAGTTGCAAATTTGGACCATCATTTTAAATCATCTGTTGGAGTTGAGC
TTTTTTCAAAACTCCAAAACACATTTTTTGGCGGTCCAAATTTTACGTCTCCGATTTTGGACCACCAAATTTTAGACCATCTATTGAAATTGAAGAT
GCTCTAACCAAAAGCGGAGCGTCCCTTTGGGTCTTCGCTTGCTCTCAGGCACCCATGTTGCGCACGGCGATCGCCGCCGCGCCGCGGCCGCGCATCT
CCCTTGCCCCGGCAAGATTCGCACACGAGCTCGCGCAGGCGCAGGCGCAGCCGGCCGTCCCTGGACGGGATGACCTTGACCCGTCGTGGCCCCCGCT
CTACAGGAGAATCTCGAGGCTGTACGGCAGGCCACCGGGAATGATGGCGGCGGAGATGGACAACTATCTCCGCAAGAGGCGCCCGCTCTCGGCGGAC
CAGATCGTGGCCTACGTCCGGAAGCTCCGCAAGTTCAAGAGCAACGCTTGCGCGCTCGAGGTGAGCCCCTACCCCCCAATCTCCCCGCAAAATGGTG
GCCTGATTTGGATCGGGATGCGTATTTGTGCCGAAAGTAGTAGGTTCGGGATAAAGCGAGTAGCGAGGAAAGGGGATGGATTGACATACTAGGGTAT
TCATTGAAAGAGCTCCATTTTGCATTCTCTTTGTGGATAATTTTAGTTTAAGGCCACTATTGGATAATTGTGCATCCTGCATTCTCGACTGAAGAAT
AGGATTGTGGTCAACTGATTGTGTTGCCAACAACAACAATGGTAATTTGTTGTCTGACGCTAAGTGAAACCAGGTGCCACACTGCACATTGCTGCAG
CTTCAGAGGGCAACACTATTAGGTAGGAAGGGCATTGGATATCCTATACTAACTATAATCGCAAAATCAGGTGATATATCGTGGCGCTACCTGTTCT
GCCTGGGAACTTTGAGGATATGTTGCAGCTTCCTTTTCTTTCTAAATATTATAGCTCCTTAGTTTTCACTTTTCGTAGTCTGAAAAGATGATTGTGT
ATAAGATGTCTTTAGCCTCAGACTTAGCGCATTCAGGTGAATTTTACTAAAGTGGCTTAGTAAATAAGGGATTGGCTGGATGTACGTCGCCTGAAAA
TCAAATTTGGGTCAGTCGATTCCATGTGAGTCGTCGGATGCAAGATGGGCAGCCAGATCAGCTTCTTCACCTCCGAAACCTTCACTGTTCATCGTCT
TCAACCTCTAACCTCTCACCCAAATTTGATTTTTCAGGCGACTTACAAGTAATTACTGTGAATAAATAAACATTGAAAACTTGGACTTAATGACCAC
AGTGAGTCAGTGACTTTAGGTCCATTACGCGTGTTGATTTAGTGCATGTGCATCCATCCAGGTGCTGACGTGTAGAGGAGAGAAGCAGATGGTTGTA
TTTGAGAGGGATTGACAGCCAGCCCTGTTGTTATTTTGGTATAAGGGTGATGCTTTGTAAAACAGATAGGCCACAAATGCTGGGCTGATAAGAAACA
TGTCAACAGTCAATGTACTGGTAGTTTTCTATACGAAACCCTGGATTTTCTGTATTTACACAAAAAGTAGCAACTGGTGGGTGTTTGCTAAATTATA
CTCCCTCGATCCATATTAATTGTCGAAGTTGTACTAAATCAGCGACAATTAATATGGATCAGACGGAACAAGCTTCAGTTGCTTTTGGTTGAAATTG
AACAATTTATGGATCTCAGGAATCAGCCAATTGTAGGCAGATACAAAGTCCAAACAAGTGAGCGGCCCTATGGCCAGGAGGAGCTCTGAAGATTGGA
GAACAAAGGACATGATAGAAGTTGGATGTTCTCCACAAGCTCAGAATGTCTCATCAAACTACAGCCCTCCAGTTATTTATAGCAGGGGGTGTCCTAG
GGCTCCTTCACATTCTCACAAATATCTGGTGGCACTATCGCCTATCTGTACTTTCCATTTCTTGGTGTCTGATTTCAGTGGTAAGATTGGGGACCTT
TTTGTCGTATCATGAGTTAATTTCCAATTTCGGTGATTAATCTGCCATTGTTAGCTTACCTGTAACACACTCTACTGCTAAGTCGGGGATCTCAATT
CTCACACATAAATAGGCAAAAACCTGGAGAAATAATGTTCTAGAGGGAAGGAAAGTAAGAATTCACAATAGGAAATGAAGGTTCAAGTTCTAACAGT
GGAGGTTTAAATTGTGATGGCATTAATGCGTCTCTCCTAAGTATCTTTTTTATCTGTAGAGATTTCATATGCTGTTCTGGTGCAACCTCTTTCTGAG
CAGAGTATTTTTTGTGTTCACTGTGTAATAACCAAATGTGTCGTTTTGTACCCAGTTGATGGACTGGATGGAATCTCGTGGAGCAAAGTTGACGCTT
GAGCATCAAGCCCTGCGGCTTGGCCTTGTTTCAAAAGTGCACGGGATACAGGCGGCTGAAGAATACTTCTGGAGCCTTCCAGATAAGTCAAAGGAAA
CTTATGTATGTTTGCTTAACTGCTATGGTGAACACGGAATGGCATGCAAAGGTCTGGAACTCTACGAGAAGATGAAAGCCATGGACATTGTTCCAGA
TACACTGGTGTACAACAGCTTGATGACTTTGTACCAAAAGGCGGACCAGCCAGAGAAAATCCCCTCCACATTTGAAGAAATGCGAGAAAGCGGTATA
AGTGCTAACAATTTTACGTACTTCACTTTGATAGAAAGCTATATCAGAATGAATGACCTGGAGGCTGCAGAGAAAGTCCTGGAAGAATTGGAGAAGG
TGGCTCCAGTTCACTGGTCCTTGTACACTTTGATGGCAAACAGTTATATTAAACTAGAGCTGTTTGGAAAGTCAGAAGTGGCCCTAAAGAAAGCTGA
GGAAGTCATGGACAAAGCTGAGTTGTTGTCCATTCTGGCAATTCAACCAAGGTTAAGAGGATCTGGGAGTCTTTTAAGTCCACATTCAAGAAATGCT
TGAACAAAAGCTATCTTGTGATGCTTGAAGCTCTGAGTATGATTGATGACTTTTGCGTCTTTGCAGCAAATCTTTCAGGAGTGGGAATCGAGTAATG
AACATTATGACATGTGGATACAAATGTTATGATCAAAGCTTACCTTGATAAGGGTATGATAGACGAAGCTGAGGCTATCCGTCATGTCTCAGGGTCA
TTGCAACGGGAGGACGGTTTACATATTTGCCGAGTTCTATTTGGACAAATCTCATGTTACCGCGGCATTGGAGATCTTGAGAGATGCAAAGAAGATG
CTTACGGCACACAAATGGGTGCCATCGGAAAAGCTTACGAGCAGATTTCTGAAGCACTATGAAGAGTCAAAGGATGTTGATGGTGTGGAGTCTTTCT
GCGAGTGCCTGAAGAAGCTCGAATGCCTTGATGCGGAAGCTTATGAGGGCATTGTGCGGACCTATATAGCTGCAGGTAGAACCAACCCATCCATTGC
TCAGCGCATCGAAGATGATGGGATTCACGTAGGACCTGAGACGACGAAATTGCTGGAGAGTGTATCCGGCAATTCAAAATGAGTAGGTAAGAAACTT
TGTTAGAAGTCGAGGCCACCATATACATTTTCTTGGTTATTATAGAGCAGAATTATGACTTGTCGAAGATGCCGTTATCTCGTGAAAAGCCCGGGAG
GAAGTATCTACTCTACAAGAATTTGATGGATCTTTATCATCCCTCGCTGTCACCGGATTTCTGTATTATCTACATTTCTAGGGCCTTTGGTATTTTA
CAT
```

Supplementary Figure 8. Genomic DNA sequences of *TraesCS6A01G091200*. Primers used to isolate this gene are highlighted in green. The start codon, stop codon, GT and AG of each intron are shown in red letters and with highlighted in yellow. No SNP was observed in the sequenced region between Duster and Billings.

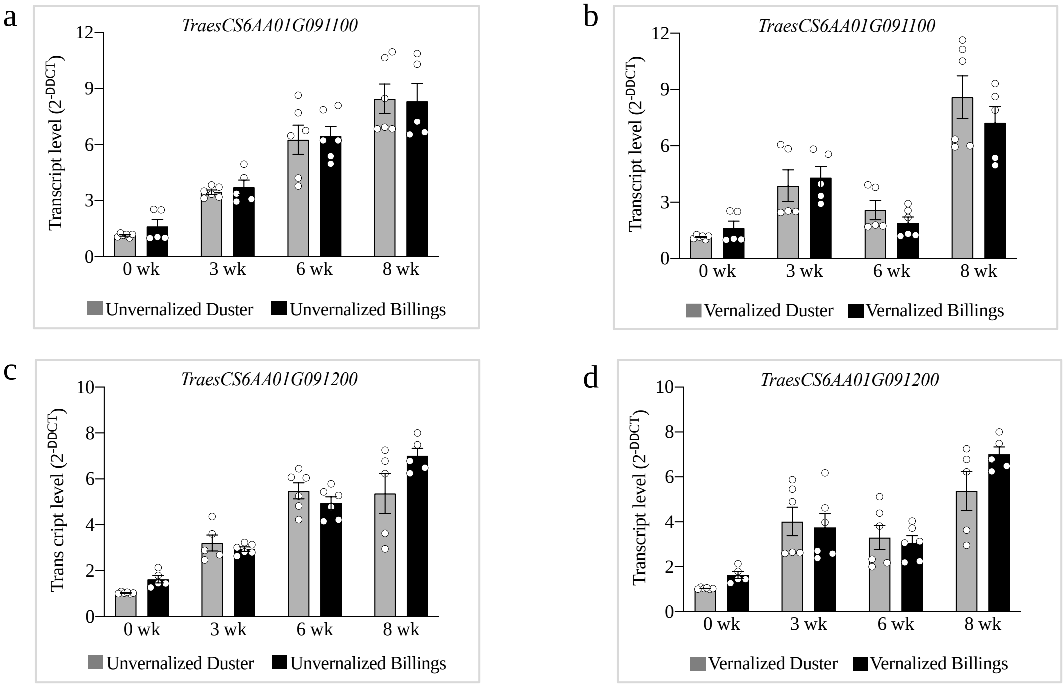

Supplementary Figure 9. Comparison of gene transcript levels of *TraesCS6A01G091100* and *TraesCS6A01G091200* between Duster and Billings. qRT-PCR was used to determine transcript levels of *TraesCS6A01G091100*, using primers 6A-100-Q-F1 (5'-TCTTTCTGCGAGTGCCTGAA-3') and 6A-100-Q-R1 (5'-CTTCTCAGTTTCAATTGCCGGAA-3'), in seedling leaves without vernalization (a) and *TraesCS6A01G091100* with vernalization (b). The cDNA samples were used to determine transcript levels of *TraesCS6A01G091200*, using primers 6A-200-Q-F1 (5'-CCAGATAAGTCAAAGGAAACTTATGT-3') and 6A-200-Q-R1 (5'-GTATCTGGAACAATGTCCATGG-3'), in the seedling leaves without vernalization (c) with vernalization (d). (a-d) Gene transcript levels are described using mean ± SEM calculated by the $2^{(-\Delta\Delta CT)}$ method, where CT is the threshold cycle. Five or six samples were tested for each of treatments with no vernalization (0 wk), 3 week-vernalization (3 wk), 6 week-vernalization (6 wk) in cold room and plants in greenhouse (GH) without vernalization for controls. Two-tailed unpaired *t* test showed no significant difference in most of the samples from the two alleles of either *TraesCS6A01G091100* or *TraesCS6A01G091200*. No difference was found in the gene sequences in either of the two genes between the two alleles. In contrary, significant difference was found in sequence and transcript level of *TraesCS6A01G091300* between the two alleles. Therefore, it was concluded that it was not *TraesCS6A01G091100* or *TraesCS6A01G091200* but *TraesCS6A01G091300* was the final candidate gene causing *QHd.osu-6A*. Source data data are provided as a Source Data file.

>*TaOGT1a*-Duster

CACAAGGAAAGGAAAGCCGGCCAGCTCGGCTGGGTTCGGTTTTACTTTACTTTGTCCCTCCTCCAAACCACGCACGCACACTGACAAGGTGACACCTCTCTCTCTCTCTCTCTCTCTCTCTCTCTCTCT
*ACCTCCCTCTCACACAAAAGAGAGAGAGAGAGAGAGAGAGAGAGAGAGAGGG*ACAGGGTGGGGGTGTTCCGTTGATTCTCCTTGGCTTGTGATTGAGAAATGAGAGGTTCGGTTCATACTTACTACAGTGGAT
TGGTAATAAAGAAGACCAATTTTAGTTAACAATGGCGTGTGCCGTGCCCCGTCCCATCTCATGGCGTACGACCCGGTGAGGTGGGGTGCGTAGGAAGGATGAGGAGGATGGATGGACAAGCAAGCGCCG
TAGATGCCCTCGCCGTCGACGCGTTGCACGTATATATATCTACCCCACGAGGAAGAAGAACAAGATAGATCGATCTCATTCCCCTTCACCTCCGCTCCGGTGCAAAGGAAAGGAAAGGGTGGAGGGAAT
AAGCAAGGCATCTCATTCCCCCCTAGCCAGCCCCACGCCTCGCCACCTTGCCGCCGCTCTCCTCCTCTCTTCTTCTTCCTCTTCACCTTTATCTCCCCCCGCGTCCAAGAATCTATCTCCTTCCCCTGT
CCTCACCTCCATTAATCCATCCCCTCCTCACATTCCCCTTCCTTGCGTGCGTACACCGTTTGGTGCTGGTACACAGATTGTTTGTTCGTTTCTTCCATGGTTGGTTGCTTCTTTGAGCGAGCGAGCGAG
TGAGAACGACGAAGAGAGGAGGCGGGGACAAGCCCGGCCAGGCACATCTATCCTGTGACCCCAAGCTTGGCATCTCCCCACACCCACGCCACACCCACACCCACACTGTTTCTTCTTCCCCTCCGCCGC
CCTGCCCTCCCCCTGCATGCCACTCGCGTGCCGCCCCGGTCCGTTGGTTGGTTGCAACGACGCCTCCTCCCCCTGCAGCATTGTGCCCGCCCTTCGTCTTCTTTAGCCTGAGCCCGGACGCGCACAAGC
AGAGCGCCGCTCTGCGACCCCGCATTGCCGCAAGAAACGCGCCGCAGAGATTGCGTCTCCTTCTTCTATCCGTAAGGCGGCAGCGCATGCCCGCCGGGACGGACTGACGGCGGCGGGGGGAGTGGCGGC
GCGGGCGATGCGGTTCACGGCGGGGGGCTCGTCGACGCGGTCGTGGCAGCCCAAGCCGACGGCGGACACCACGGACCTGCGCTTCTGGCTGCGCTGGCGGGTGGCCGTGTGCGCGCTCTGGGTGCTCTG
CTGCGTCGCCGCCGCCGCCTACCTCGTCTGGCGCCACGAGGGGCCGCGCGCGCACCGCCGCCCCGGCGCCGCCAAGCAGGACGCGGCCGCGCAAGGGCGGCGGCGCCCGGACGGGCTGCTCTACGACGA
CGAGGCGTGGCGGCCCTGCCTCCGCGACATCCACCCGGCCTGGCTGCTCGCCTACAGGCTCGTCTCCTTCTTCGTCCTCTTCAGCCTCCTCATCGTCATCGTCATCTCCGACGGCGGCAACATCTTCTA
CTACTACACCCAGTAAGCCTTCCTCCACTCCACTCACCCACCACCATCAGCATCATCTACCACTGCTTGCTCCTTTTTTTACTCTGTTTCCGAAGAAAGATTTTTTTTACTTGGGTGATGACTGGCCGT
AGATGCAAAGTTGCATTAAAGGTTTATGCTTTGGCATTTCCCATTTGATTAAAATCCTTGAGGCTGTAAAATTGACTGGAGTGGCAGCCAGCAATGCATATGGTGTGCCCAGCAGCAACTTTGGCATCT
GTTCTCTCTTGTTGTGAATTTCAATTTTATCTCTGGGGAAGGGAATAAAGGGAGGCTTCTTCTCATGATTACATCAACTACTGATCTTAGACATGTTTCCTCAATAAACTAAGAGTCAGTTTTATTCAT
GATGCGCTACTTCATTTTTAATATTTTGAAATTGCCTCTCACAGGACATGTTCTGTCAAGCACATCAGTGAAACCAATATTTTTCTTGGGGGCAGTGTTGATTTTCATTTTCACTGGTATTTATTTTGG
GGCATCAATGCATTGTAGAGATAGTCTTGATGGATGCAGTGCTTCAATTTAATAATCAGTAAGGACCTCATATACATGCTTTGTCAAGCACATTGAAGGAACAATCTTTTTTGTGCAGTACAATGTCAC
ACAAGCAGGACAGTGCTGTGGGAATACCTGTTCCATTGAGGATTTCTGACACCAGATTAGCTTAGCAGCCCCCATTTTGATACCATTACTTTTCTGGGGGGCAAATCTCCAGTGTGCTTTTTTCTTTTC
CTCCAAAGGAAAAGAAAATGTGGTGTGTAACTGGTTGGTAGCATCAAAGCTGTCTGCAAAGATGTGATACTACTTGCTGTTGTTGGTGTCTGTGACTCTGCTCTCAGTAAGCAGCATTTGTCCCTAGCT
TGCACGTACTAAGTGGTTGTTCAACTGTCTATTACGCAGGTGGACCTTCATTCTGGTCACGGTTTACTTCGGGGTAAGCGAGAAATAGCCTGTCTCCTCACTGATTTCTTGGTGCACAATGATGAGCGT
CCCCACGGCCTCTATCAGTGTCCTCACAGTCCTTATCTGAAGAGACTATATGCGGTTCTGTGATTTCCCCAGCTTGCGACGACGCTCTCGATCTACGGGTGCAGCAAGTTCGCCGCGTGCAATGCCGTC
GCGGCGATGTCCGACGCCGAGCAGGGGCCCTACGCCGTCCACGGGGCCGCCCCGAAGCCGGTCGTCGACAGGGAGGACGACGGCACGAGGGAGGTCGCCGGGTTCTGGGGGTACCTGCTGCAGATCATC
TATCAGGTAAAGGAGGTTTTTTCATGCCACCTATAACCTTTGAAATGTTTGGCCAACATTGAGCCCGTTTTCCAGATTTCATATTGGAGAGGGACATCTGCACTGACATCACATCTTCATTGCATTTGC
ATTTGCATTTGCATGGTGGGGTGGTCTCAGTTTTTGCTTTTCAGGTGCAATTATTAGTGCTCCTGGGTAGTTAACATCATGCACCAGAAACTAAAGTGTGTTTATAATGTGCTCTGCTGAATTGGATTC
CTGTGCATCAAAGATGGGGATGTGACCACATACTAGCATCTTTGTCTGGCAGATCCTACATGAGCCACCTTCTTGGTTGTTGGGCTGGACCCAAATGCCCTATCTCTTAGTAGTTCCTTGCACCTGAAG
GCCTTGCTTGATCACCTTTCTTTACCCCATATCTCAAGTGCAGGGCTGGCTTTTTTAGCTGTAGCATCAGGAATAAAGACAAAATTTTGTTTATTTTTCTCTGTTGATTTGCTAGGCCTGACTGTATCA
GAATTATGTCTGGATATCTCTCTAGGCTTGGATAAATTATAGACCAGGGGTAGTGGATATTTCCACTGATAATAACCATAGTACAGCTTCCAGTAGTATTACAGTAGAAAAATGGGAGAAAAATATTAC
GACAAGTGATGTAATTGGCAACTTATGCAAGACAGTTGTTATCATTTTACTGCCAATTCACCAAACTATTATTCTGACGTCTCCATCTCTGTTGACAGACAAATGCAGGGGCCGTGATGCTTACAGACT
GCGTCTTCTGGTTCATCATTTTCCCATTCCTCACCGTAAAAGATTACAGCATGAATTTTGTTAGTGTGTTTGTCAATTTCTTGGCTTTTGTATTGGTCATCTGATTGATGATAATCAGTTTGCTAACTT
GATCCTTGTCTGACAGCTATTGATAGGAATGCACTCGGTCAACGCTGTTTTCTTGCTCGGCGAAGCATCGCTAAATAGCCTGGTAAGTGGATGAACACTCAACTTGATCACCAAAGCAGGTTGATGCAT
GGTTTTTACAGTTTACTCGCCGCATCTGATTTTAACTTCACACAACTTTTACAGCGCTTCCCGTGGTTCCGGATCGCGTATTTCTTCCTTTATACGGCGCTATACGTAGTTTTCCAGTGGATTGTTCAT
GCATCTACTCCAACCTGGTAAAATCCCCAGCTTCCATTCCCTCCCTAGAGCAAAATACTGTCTGCATATTATCTTTCTGTCACTGAACATATGTCATGTGAATTCTGGCAGGTGGCCCTACCCGTTCCT
CGACCTCTCCTCTAATCTCGCGCCTCTGTGGTAATTCCATTTACCACCCTATAACAGCAATTATTTCCCTGCTTTGTTTGACGTAGATTTTATTAACTGAAGTATGTTTGATCACAAGCTGAGCCTGAA
CTTATTCCATAGGTACTTTGCGGTCGCGTTCATGCAACTGCCCTGCTACTTGATCTTCAGACTGGTGATGAACCTGAAACACCACCTTCTCGCCAAGCATTTCCCGGATAGCATGGTCCTAGGATACTA
GCATCCGTCAATGAACATCCATGCCGGTCTTCTTCCTTCGATCCTCCACGCAAAAAGGCGATGCGAATTGGCAGGAAGAATAACAACTGGACAATGGCGGCGTGGCAGTAGATGTTCTAGAA

>*TaOGT1b*-Billings

CACAAGGAAAGGAAAGCCGGCCAGCTCGGCTGGGTTCGGTTTTACTTTACTTTGTCCCTCCTCCAAACCACGCACGCACACTGACAAGGTGACCTCTCTCTCTCTCTCTCTCTCTCTCTCTCTCTCT
*CTCTCTCTCTCTCTCTCTCTACGGCCAGCTCAGCCAGCAACAGAGAGAGAGAGAGAGAGAGAGAGGG*ACAGGGTGGGGGTGTTCCGTTGATTCTCCTTGGCTTGTGATTGAGAAATGAGAGGTTCGGTTCA
TACTTACTACAGTGGATTGGTAATAAAGAAGACCAATTTTAGTTAACAATGGCGTGTGCCGTGCCCCGTCCCATCTCATGGCGTACGACCCGGTGAGGTGGGGTGCGTAGGAAGGATGAGGAGGATGGA
TGGACAAGCAAGCGCCGTAGATGCCCTCGCCGTCGACGCGTTGCACGTATATATATCTACCCCACGAGGAAGAAGAACAAGATAGATCGATCTCATTCCCCTTCACCTCCGCTCCGGTGCAAAGGAAAG
GAAAGGGTGGAGGGAAGAAGCAAGGCATCTCATTCCCCCCTAGCCAGCCCCACGCCTCGCCACCTTGCCGCCGCTCTCCTCCTCTCTTCTTCTTCCTCTTCACCTTTATCTCCCCCCGCGTCCAAGAAT
CTATCTCCTTCCCCTGTCCTCACCTCCATTAATCCATCCCCTCCTCACATTCCCCTTCCTTGCGTGCGTACACCGTTTGGTGCTGGTACACAGATTGTTTGTTCGTTTCTTCCATGGTCGGTTGCTTCT
TTGAGCGAGCGAGTGAGAACGACGAAGAGAGGAGGGCGGGGACAAGCCCGGCCAGGCACATCTATCCTGTGACCCCAAGCTTGGCATCTCCCCACACCCACGCCACACCCACACCCACACTGTTTCTTC
TTCCCCTCCGCCGCCCTGCCCTCCCCCTGCATGCCACTCGCGTGCCGCCCCGGTCCGTTGGTTGGTTGCAACGACGCCTCCTCCCCCTGCAGCATTGTGCCCGCCCTTCGTCTTCTTTAGCCTGAGCCC
GGACGCGCACAAGCAGAGCGCCGCTCTGCGACCCCGCATTGCCGCAAGAAACGCGCCGCAGAGATTGCGTCTCCTTCTTCTATCCGTAAGGCGGCAGCGCATGCCCGCCGGGACGGACTGACGGCGGCG
GGGGGAGTGGCGGCGCGGGCGATGCGGTTCACGGCGGGGGGCTCGTCGACGCGGTCGTGGCAGCCCAAGCCGACGGCGGACACCACGGACCTGCGCTTCTGGCTGCGCTGGCGGGTGGCCGTGTGCGCG
CTCTGGGTGCTCTGCTGCGTCGCCGCCGCCGCCTACCTCGTCTGGCGCCACGAGGGGCCGCGCGCGCACCGCCGCCCCGGCGCCGCCAAGCAGGACGCGGCCGCGCAAGGGCGGCGGCGCCCGGACGGG
CTGCTCTACGACGACGAGGCGTGGCGGCCCTGCCTCCGCGACATCCACCCGGCCTGGCTGCTCGCCTACAGGCTCGTCTCCTTCTTCGTCCTCTTCAGCCTCCTCATCGTCATCGTCATCTCCGACGGC
GGCAACATCTTCTACTACTACACCCAGTAAGCCTTCCTCCACTCCACTCACCCACCACCATCAGCATCATCTACCACTGCTTGCTCCTTTTTTTACTCTGTTTCCGAAGAAAGATTTTTTTTACTTGGG
TGATGACTGGCCGTAGATGCAAAGTTGCATTAAAGGTTTATGCTTTGGCATTTCCCATTTGATTAAAATCCTTGAGGCTGTAAAATTGACTGGAGTGGCAGCCAGCAATGCATATGGTGTGCCCAGCAG
CAACTTTGGCATCTGTTCTCTCTTGTTGTGAATTTCAATTTTATCTCTGGGGAAGGGAATAAAGGGAGGCTTCTTCTCATGATTACATCAACTACTGATCTTAGACATGTTTCCTCAATAAACTAAGAG
CAGTGAGAATCTGCAACAAATTTCCTTGCATTCATATTGATCTTCTGTGCACAAGTGATGGCTGGCAGCAGTTTGCTAGTAGTACCTAGCTTCCACAGCAAGCCACTCCTCAACTGTTGGGTTTCATTT
TCAGTTGTACATTTTTTTGGGTGTCAATGCATTTTAGAGTCAGTTTTATTCATGATGCGCTACTTCATTTTTAATATTTTGAAATTGCCTCTCACAGGACATGTTCTGTCAAGCACATCAGTGAAACCA
ATATTTTTCTTGGGGGCAGTGTTGATTTTCATTTTCACTGGTATTTATTTTGGGGCATCAATGCATTGTAGAGATAGTCTTGATGGATGCAGTGCTTCAATTTAATAATCAGTAAGGACCTCATATACA
TGCTTTGTCAAGCACATTGAAGGAACAATCTTTTTTGTGCAGTACAATGTCACACAAGCAGGACAGTGCTGTGGGAATACCTGTTCCATTGAGGATTTCTGACACCAGATTAGCTTAGCAGCCCCCATT
TTGATACCATTACTTTTCTGGGGGGCAAATCTCCAGTGTGCTTTTTTCTTTTCCTCCAAAGGAAAAGAAAATGTGGTGTGTAACTGGTTGGTAGCATCAAAGCTGTCTGCAAAGATGTGATACTACTTG
CTGTTGTTGGTGTCTGTGACTCTGCTCTCAGTAAGCAGCATTTGTCCCTAGCTTGCACGTACTAAGTGGTTGTTCAACTGTCTATTACGCAGGTGGACCTTCATTCTGGTCACGGTTTACTTCGGGGTA
AGCGAGAAATAGCCTGTCTCCTCACTGATTTCTTGGTGCACAATGATGAGCGTCCCCACGGCCTCTATCAGTGTCCTCACAGTCCTTATCTGAAGAGACTATATGCCGTTCTGTGATTTCCCCAGCTTG
CGACGACGCTCTCGATCTACGGGTGCAGCAAGTTCGCCGCGTGCAATGCCGTCGCGGCGATGTCCGATGCCGAGCAGGGGCCCTACGCCGTCCACGGGGCCGCCCCGAAGCCGGTCGTCGACAGGGAGG
ACGACGGCACGAGGGAGGTCGCCGGGTTCTGGGGGTACCTGCTGCAGATCATCTATCAGGTAAAGGAGGTTTTTTCATGCCACCTATAACCTTTGAAATGTTTGGCCAACATGGAGCCCGTTTTCCAGA
TTTCATATTGGAGAGGGACATCTGCACTGACATCACATCTTCATTGCATTTGCATTTGCATTTGCATGGTGGGGTGGTCTCAGTTTTTGCTTTTCAGGTGCAATTATTAGTGCTCCTGGGTAGTTAACA
TCATGCACCAGAAACTAAAGTGTGTTTATAATGTGCTCTGCTGAATTGGATTCCTGTGCATCAAAGATGGGGATGTGACCACATACTAGCATCTTTGTCTGGCAGATCCTACATGAGCCACCTTCTTGG
TTGTTGGGCTGGACCCAAATGCCCTATCTCTTAGTAGTTCCTTGCACCTGAAGGCCTTGCTTGATCACCTTTCTTTACCCCATATCTCAAGTGCAGGGCTGGCTTTTTTAGCTGTAGCATCAGGAATAA
AGACAATATTTTGTTTATTTTTCTCTGTTGATTTGCTAGGCCTGACTGTATCAGAATTATGTCTGGATATCTCTCTAGGCTTGGATAAATTATAGACCAGGGGTAGTGGATATTTCCACTGATAATAAC
CATAGTACAGCTTCCAGTAGTATTACAGTAGAAAAATGGGAGAAAAATATTACAACAAGTGATGTAATTGGCAACTTATGCAAGACAGTTGTTATCATTTTACTGCAAATTCACCAAACTATTATTCTG
ACGTCTCCATCTCTGTTGACAGACAAATGCAGGGGCCGTGATGCTTACAGACTGCGTCTTCTGGTTCATCATTTTCCCATTCCTCACCATAAAAGATTACAGCATGAATTTTGTAAGTGTGTTTGTCAA
TTTCTTGGCTTTTGTATTGGTCATCTGATTGATGATAATCAGTTTGCTAACTTGATCCTTGTCTGACAGCTATTGATAGGAATGCACTCGGTCAACGCTGTTTTCTTGCTCGGCGAAGCATCGCTAAAT
AGCCTGGTAAGTGGATGAACACTCAACTTGATCACCAAAGCAGGTTGATGCATGGTTTTTACAGTTTACTCGCCGCATCTGATTTTAACTTCACACAACTTTTACAGCGCTTCCCGTGGTTCCGGATCG
CGTATTTCTTCCTTTATACGGCGCTATACGTAGTCTTCCAGTGGATTGTTCATGCATCTACTCCAACCTGGTAAAATCCCCAGCTTCCATTCCCTCCCTAGAGCAAAATACTGTCTGCATATTATCTTT
CTGTCACTGAACATATGTCATGTGAATTCTGGCAGGTGGCCCTACCCGTTCCTCGACCTCTCCTCTAATCTCGCGCCTCTGTGGTAATTCCATTTACCACCCTATAACAGCAATTATTTCCCTGCTTTG
TTTGACGTAGATTTTATTAACTGAACTATGTTTGATCACAAGCTGAGCCTGAACTTATTCCATAGGTACTTTGCGGTCGCGTTCATGCAACTGCCCTGCTACTTGATCTTCAGACTGGTGATGAACCTG
AAACACCACCTTCTCGCCAAGCATTTCCCGGATAGCATGGTCCTAGGATACTAGCATCCGTCAATGAACATCCATGCCGGTCTTCTTCCTTCGATCCTCCACGCAAAAAGGCGATGCGAATTGGCAGGA
AGAATAACAACCGGACAATGGCGGCGTGGCAGTAGATGTTCTAGAA

Supplementary Figure 10. Comparison of *TaOGT1* gDNA sequences between the Duster and Billings alleles. The reference of CS is *TraesCS6A01G091300*. The italic sequence of the first 177 bp was poor in quality due to the presence of simple repeat sequence. The start codon, stop codon, GT and AG of each intron are shown in red letters and with highlighted in yellow. Different SNPs in the region between the start codon ATG and the stop codon TAG are indicated with highlighted in red. One nucleotide in coding region that caused amino acid to change is highlighted in purple. Different sequences in the 5' and 3' ends of the gene are highlighted in green.

```
a.>NP_201101.2 UDP-N-acetylglucosamine-N-acetylmuramyl-pyrophosphoryl-undecaprenol N-
acetylglucosamine protein [Arabidopsis thaliana]
MVSSSSSLLSPSSYTELKDAWHPSTTANTTESSYWFNWRVMICCIWMAIATVITAFLIFKYEGFRRKRSDVGEVDGGEKEWSGNVYEDETWRPCLRN
IHPAWLLAFRVVAFFVLLVMLIVIGLVDGPTIFFYYTQWTFGLITLYFGLGSLLSLHGCYQYNKRAAGDRVDSIEAIDSERARSKGADNTIQQSQYS
SNPAGFWGYVFQIIFQMNAGAVLLTDCVFWFIIVPFLEIHDYSLNVLVINMHSLNAIFLLGDAALNSLSFPCFRIAYFFFWTIAYVIFQWALHSLVH
IWWPYPFLDLSSHYAPLWYFSVAVMHLPCYGAFALLVKLKHRLLQRWFPESYQSPR
```

b.>Comparison of *Ta*OGT1 with orthologous protein in Arabidopsis.

```
Query  12   SWQPKPTADTTDLRFWLRWRVAVCALWVLCCVAAAAYLVWRHEGPRAHRRPGAAKQDAAA  71
            +W P  TA+TT+  +W  WRV +C +W+       A+L++++EG R  +R    + D
Sbjct  20   AWHPSTTANTTESSYWFNWRVMICCIWMAIATVITAFLIFKYEGFR-RKRSDVGEVDG--  76

Query  72   QGRRRPDGLLYDDEAWRPCLRDIHPAWLLAYRLVSFFVLFSLLIVIVISDGGNIFYYYTQ  131
             G +   G +Y+DE WRPCLR+IHPAWLLA+R+V+FFVL  +LIVI + DG  IF+YYTQ
Sbjct  77   -GEKEWSGNVYEDETWRPCLRNIHPAWLLAFRVVAFFVLLVMLIVIGLVDGPTIFFYYTQ  135

Query  132  WTFILVTVYFGLATTLSIYGC---SKFAACNAVAAMSDAEQGPYAVHGAAPKPVVDREDD  188
            WTF L+T+YFGL + LS++GC    +K AA + V ++   +       GA       ++
Sbjct  136  WTFGLITLYFGLGSLLSLHGCYQYNKRAAGDRVDSIEAIDSERARSKGADNTI---QQSQ  192

Query  189  GTREVAGFWGYLLQIIYQTNAGAVMLTDCVFWFIIFPFLTIKDYSMNFLLIGMHSVNAVF  248
             +   AGFWGY+ QII+Q NAGAV+LTDCVFWFII PFL I DYS+N L+I MHS+NA+F
Sbjct  193  YSSNPAGFWGYVFQIIFQMNAGAVLLTDCVFWFIIVPFLEIHDYSLNVLVINMHSLNAIF  252

Query  249  LLGEASLNSLRFPWFRIAYFFLYTALYVVFQWIVHASTPTWWPYPFLDLSSNLAPLWYFA  308
            LLG+A+LNSL FP FRIAYFF +T  YV+FQW +H+    WWPYPFLDLSS+ APLWYF+
Sbjct  253  LLGDAALNSLSFPCFRIAYFFFWTIAYVIFQWALHSLVHIWWPYPFLDLSSHYAPLWYFS  312

Query  309  VAFMQLPCYLIFRLVMNLKHHLLAKHFPDS  338
            VA M LPCY  F L++ LKH LL + FP+S
Sbjct  313  VAVMHLPCYGAFALLVKLKHRLLQRWFPES  342
```

c.>*Ta*OGT1a-Duster

```
MRFTAGGSSTRSWQPKPTADTTDLRFWLRWRVAVCALWVLCCVAAAAYLVWRHEGPRAHRRPGAAKQDAAAQGRRRPDGLLYDDEAWRPCLRDIHPA
WLLAYRLVSFFVLFSLLIVIVISDGGNIFYYYTQWTFILVTVYFGLATTLSIYGCSKFAACNAVAAMSDAEQGPYAVHGAAPKPVVDREDDGTREVA
GFWGYLLQIIYQTNAGAVMLTDCVFWFIIFPFLTVDYSMNFLLIGMHSVNAVFLLGEASLNSLRFPWFRIAYFFLYTALYVVFQWIVHASTPTWWPY
PFLDLSSNLAPLWYFAVAFMQLPCYLIFRLVMNLKHHLLAKHFPDSMVLGY*
```

d.>*Ta*OGT1b-Billings

```
MRFTAGGSSTRSWQPKPTADTTDLRFWLRWRVAVCALWVLCCVAAAAYLVWRHEGPRAHRRPGAAKQDAAAQGRRRPDGLLYDDEAWRPCLRDIHPA
WLLAYRLVSFFVLFSLLIVIVISDGGNIFYYYTQWTFILVTVYFGLATTLSIYGCSKFAACNAVAAMSDAEQGPYAVHGAAPKPVVDREDDGTREVA
GFWGYLLQIIYQTNAGAVMLTDCVFWFIIFPFLTIKDYSMNFLLIGMHSVNAVFLLGEASLNSLRFPWFRIAYFFLYTALYVVFQWIVHASTPTWWP
YPFLDLSSNLAPLWYFAVAFMQLPCYLIFRLVMNLKHHLLAKHFPDSMVLGY*
```

Supplementary Figure 11. Protein sequences of *Ta*OGT1 in Arabidopsis and wheat. a. Orthologous protein in Arabidopsis (NP_201101.2). b. Alignment of *Ta*OGT1 protein in CS (Query in first line) with its orthologous protein in Arabidopsis (NP_201101.2) (Sbjct in second line). c. *Ta*OGT1a in Duster. d. *Ta*OGT1b in Billings. The 'I' in *Ta*OGT1b from Billings is replaced by 'V' in *Ta*OGT1a from Duster. The point substituted amino acid is highlighted in red.

```
>The regulatory region-Duster
AGGCACATCTATCCTGTGACCCCAAGCTTGGCATCTCCCCACACCCACGCCACACCCACACCCACACTGTTTCTTCTTCCCCTCCGCCGCCCTGCCC
TCCCCCTGCATGCCACTCGCGTGCCGCCCCGGTCCGTTGGTTGGTTGCAACGACGCCTCCTCCCCCTGCAGCATTGTGCCCGCCCTTCGTCTTCTTT
AGCCTGAGCCCGGACGCGCACAAGCAGAGCGCCGCTCTGCGACCCCGCATTGCCGCAAGAAACGCGCCGCAGAGATTGCGTCTCCTTCTTCTATCCG
TAAGGCGGCAGCGCATGCCCGCCGGGACGGACTGACGGCGGCGGGGGGAGTGGCGGCGCGGGCGATGCGGTTCACGGCGGGGGGCTCGTCGACGCGG
TCGTGGCAGCCCAAGCCGACGGCGGACACCACGGACCTGCGCTTCTGGCTGCGCTGGCGGGTGGCCGTGTGCGCGCTCTGGGTGCTCTGCTGCGTCG
CCGCCGCCGCCTACCTCGTCTGGCGCCACGAGGGGCCGCGCGCGCACCGCCGCCCCGGCGCCGCCAAGCAGGACGCGGCCGCGCAAGGGCGGCGGCG
CCCGGACGGGCTGCTCTACGACGACGAGGCGTGGCGGCCCTGCCTCCGCGACATCCACCCGGCCTGGCTGCTCGCCTACAGGCTCGTCTCCTTCTTC
GTCCTCTTCAGCCTCCTCATCGTCATCGTCATCTCCGACGGCGGCAACATCTTCTACTACTACACCCAGTAAGCCTTCCTCCACTCCACTCACCCAC
CACCATCAGCATCATCTACCACTGCTTGCTCCTTTTTTTACTCTGTTTCCGAAGAAAGATTTTTTTTACTTGGGTGATGACTGGCCGTAGATGCAAA
GTTGCATTAAAGGTTTATGCTTTGGCATTTCCCATTTGATTAAAATCCTTGAGGCTGTAAAATTGACTGGAGTGGCAGCCAGCAATGCATATGGTGT
GCCCAGCAGCAACTTTGGCATCTGTTCTCTCTTGTTGTGAATTTCAATTTTATCTCTGGGGAAGGGAATAAAGGGAGGCTTCTTCTCATGATTACAT
CAACTACTGATCTTAGACATGTTTCCTCAATAAACTAAGAGTCAGTTTTATTCATGATGCGCTACTTCATTTTTAATATTTTGAAATTGCCT

>The regulatory region-Billings
AGGCACATCTATCCTGTGACCCCAAGCTTGGCATCTCCCCACACCCACGCCACACCCACACCCACACTGTTTCTTCTTCCCCTCCGCCGCCCTGCCC
TCCCCCTGCATGCCACTCGCGTGCCGCCCCGGTCCGTTGGTTGGTTGCAACGACGCCTCCTCCCCCTGCAGCATTGTGCCCGCCCTTCGTCTTCTTT
AGCCTGAGCCCGGACGCGCACAAGCAGAGCGCCGCTCTGCGACCCCGCATTGCCGCAAGAAACGCGCCGCAGAGATTGCGTCTCCTTCTTCTATCCG
TAAGGCGGCAGCGCATGCCCGCCGGGACGGACTGACGGCGGCGGGGGGAGTGGCGGCGCGGGCGATGCGGTTCACGGCGGGGGGCTCGTCGACGCGG
TCGTGGCAGCCCAAGCCGACGGCGGACACCACGGACCTGCGCTTCTGGCTGCGCTGGCGGGTGGCCGTGTGCGCGCTCTGGGTGCTCTGCTGCGTCG
CCGCCGCCGCCTACCTCGTCTGGCGCCACGAGGGGCCGCGCGCGCACCGCCGCCCCGGCGCCGCCAAGCAGGACGCGGCCGCGCAAGGGCGGCGGCG
CCCGGACGGGCTGCTCTACGACGACGAGGCGTGGCGGCCCTGCCTCCGCGACATCCACCCGGCCTGGCTGCTCGCCTACAGGCTCGTCTCCTTCTTC
GTCCTCTTCAGCCTCCTCATCGTCATCGTCATCTCCGACGGCGGCAACATCTTCTACTACTACACCCAGTAAGCCTTCCTCCACTCCACTCACCCAC
CACCATCAGCATCATCTACCACTGCTTGCTCCTTTTTTTACTCTGTTTCCGAAGAAAGATTTTTTTTACTTGGGTGATGACTGGCCGTAGATGCAAA
GTTGCATTAAAGGTTTATGCTTTGGCATTTCCCATTTGATTAAAATCCTTGAGGCTGTAAAATTGACTGGAGTGGCAGCCAGCAATGCATATGGTGT
GCCCAGCAGCAACTTTGGCATCTGTTCTCTCTTGTTGTGAATTTCAATTTTATCTCTGGGGAAGGGAATAAAGGGAGGCTTCTTCTCATGATTACAT
CAACTACTGATCTTAGACATGTTTCCTCAATAAACTAAGAGCAGTGAGAATCTGCAACAAATTTCCTTGCATTCATATTGATCTTCTGTGCACAAGT
GATGGCTGGCAGCAGTTTGCTAGTAGTACCTAGCTTCCACAGCAAGCCACTCCTCAACTGTTGGGTTTCATTTTCAGTTGTACATTTTTTTGGGTGT
CAATGCATTTTAGAGTCAGTTTTATTCATGATGCGCTACTTCATTTTTAATATTTTGAAATTGCCT
```

Supplementary Figure 12. DNA sequences of the regulatory region fused with the reporter gene GUS. Primers used to amplify the regulatory region are highlighted in green. The region includes 355 bp before the start codon, exon 1 that is highlighted in gray, and partial intron from highlighted ‘GT’ in yellow. The 168-bp insertion in Billings is highlighted in blue, which is inserted between the sequence (ATAAACTAAGAG) in red and the sequence (TCAGTTTTATTC) in purple. The CArG box in the insertion is indicated in red.

>*Traes*CS1B02G364800 gDNA sequence (595,180,565:595,184,659 bp on chromosome 1B)
TCAGAGGACTCTCAGCTGGGTTAGAAAGGCAGAACACAGGTCCAGAAACAGGAGCTGTGGTCCACTGACCCTTTGCAAGTCGAGAAGATACTTCTCA
TCCCTTGTTTTGTAGAGCTGCAAAGAGCATAAGTGTATCAACACCTAGCACATCCTCAGAGGATTAACTTTATGCAAGCAAAAAGAAGTGTGATATG
AGCGAAAATAAGGTTGATTATGAACATGCCTGAATATGATACTACCTCTGTAAACAAATATAAGACGTTTTAGATCACTAAAGGTTCTATTTGTTTA
GAGGGAGTAATAGTTAACGATAATCTTACATGCAAATGCACTACTACCTCCGATCCATATTAATTGACGCAGCATCAACTAATATGGATCGGAGGGA
GTAACAAAATTAATTTCACTGCTTAGCCATCACACAAGGAGTAGAAAAACATGTCAACGCTGAGGCTTATGCATTAGATTGTTCACATAACCACATG
GCAAATTTGCAAATCAGGACCAGCTCACAAACTAACGGCAGATGGTTAAGATTAGAAGGGTACCTGAATTTCAAACTTCACGATGTGGGTTGATTTG
TCACTGAGGCCATCAGCTTCAATTATAGCAGATTCCACACCGAAGCCATTGTTGTTATGCATCATAGTCTCAACAAAGCCAGGACTCCACCTGCACT
TCATGTTATAGTGCCCAATCTTCTTCCAGCAAACATTTAGCTCTTGCAGAGCTTTAAGCACTTCAGTTATGATTTCTCGTGGATGTGCTCGAGACTG
TCAAATATCATAAAGTAAGAAAATCTAAGGATTTCGGCTTCAGTTATAGCAAGATGAATTTCAAACCAAAATTACCTGAAGACCAAGAGCCCATTTC
CTCTCAGCTGCAAAATGGTGCCTCAAGCCAAACCCTGGAGAACCATACTGCCGAGCTTCAGATGCTGAACTTGGTGTTTCAGGTGAAACTTGGGAGA
AAGATGAGTCCTGCATATCACAGAAAGCACAGCAAATGAGTAGCAGTACGAGTACAAGAGCAATTGCAATCTACAGCACAAGCTATACAAATGAGAA
AAAAAACTCCCAACACACCATTGACTCTTGATACTCGGCTCCAAGGTAGCCACTGGTTGTACGAAGCTTATTGTCCAGGAGTAAATAATATGCAACT
GTCGCCTGCACAGGTAGAATTTAGTAATTGTTCCATTAAGAACAACCAAAAAGAACAGAGTAATGCAAATACCTCATTTTGCAATCTCTTTTGAAGA
GATTCAGTGAGCTGATTCTTGTCAAATCCCATTTTGATAACATCATTCAGAGTTTCATCGTCAAGCTACAAAATTCATAGTGACGTCAAAATTACTC
CTGAACATGCATAAAGAAAGTGTAATAACGCAGAAAGGACTCCCATTTTGTACATATATGAATACACAAAGATAAAGAAAAATAAATATATTCACCA
CTGTTATAGAATGCCATAAGAGCAAAGGTTGCAAAAATCTTAAAGTTCATGTTGATACACAGCATCATCAGCATACCTTTTTGACTTGTTGTGCAGT
GTCTGGAGGAGGCACGGCCAAATAGCGTGGAAGTCTAGCTTTGAACCATGAATGTTCACGGATTTCACGTATGGTAATCCTCTTCATAGGATCAACA
ACCAGCATTCTTGGGATCAAATCTCTTGCTAAAGGAGACAAGTGACTAGGAAGGGTGTATATTCCCCCTATAATGACAAAAAAGATAACTTATTCA
CTGGATATAAGATGAAATGGTATAGCACATTTTTCAGTAAACTGAAGAAGATAGTTGCTAACCTTTATTTTCTTAAAAAGGTTTGGTATGTTCTCAT
CATCAAATGGAAGAGTGCCACAAAGAAGGGCATACAGAATAACACCACAGCTCCAAACATCAACTTCAGGGCCAGCGTACAGTTTACCTGATATCAC
CTAAGCAAATGTAGAAAACATTGTCTGGTATCAGAGTTGTGACACATTATCACAATATCAACATTTATTAACCCTGTGAAGTGTCAAACAAGCTATT
CTAGAGATATACCTCGGGTGCTGCATAATTTGGGCTACCACAACTAGTCTTCAGAAAGTGGCCGTCACGCATAACATTACTTAAGCCAAAATCTGCA
ATCTTAACATTACATTTCGAATCCAACAGAAGGTTCTCTGGCTTCAGATCACGGTGAACCACCATATTTCTATGGCAATATTCCACACCAGATATAA
TCTGTATGAAAGGTGAAACAAATAAATAGCGTACCATAAGCAGTGAATAATGTTGATACACAATGATATTATGATAGAAGCAGGCAAAAACAAAGTA
TCTACAGTGACAAATATGACTAACAAACATGACATGTACTTTATATTAGCTAATGGTAGCATACTGAATTTATAACTGTAGAATAAATCAATAAAAG
GGCTTGACCCAATCTTTGCAATGAATTCAGGGAAGTGAAAGAAACGTACTTGCTGGAAAAAGCGACGAGCTTCTTCCTCTTGTAACCTTCCCTTCTC
AACAATGTAATCAAACAACTCCCCAGATTTAACATACTCCATAACAACATAAATATCCGCTGGGGTATCTATGACCTCATAAAGTCGTATGATATGA
GGATGCATAAATAACCTCAGTATCTTGATTTCTCTTTTCACTGTAAATTGGCAAAAAAAAACTGAGAATTGAGCAAGTGCAAAACAACAATAGTGGC
AAGAATACAAGTATATGTCAATCAGCAAAGTAACAAACAGCAGCTTTTATAATGTAAAAAAATCAACAAGCTATTGATTGTGATACTTATACTAGAC
ACATCAGCCAACAGAAATTTGCAAGTTGATTACTAAAGCATCATAGGTTTATAACACAAATAAGGTTGAATAGTTTGAGAGGTTGTGCTTTCTACCT
TCTACTAGAAACATGCAAATCAAACAATGTAATCAACCAACTCTTCAGATTTAACATACGGAGTCAATCACTAGCTCGTAGCTCGAGAGCCTAATGA
GCGATCGATAGCTTGGCTAATTAAGCTCGTAGGTCACTTCTTAATGAACAAAGCCAATCACTGATTTCAGCTCGTTAAACTTAACGAGTCTCACGAC
AGCTCGTTGAGCTCGCTAGTACAACACAACACATATTCTTTTCTTAGGTGACAACATTTTCGCGACACCTCGGTCCATCTATTCAATCAAATCGACC
TAACATAGCCCATAGGAGGCAGGAAACTAGGGCACCTCCTCCTGTAGTCACCATACTCCTTCATAAAAGTCACACACACACTCTCAAAATGTAGGTC
GTAACACATCGTAGTCTGTTAACGAGCGCTCGCAAACACTCACGAGCTTAACAGAGCCTAGGAGGGATTTTTGCTCGTTAAGCTTAACGACCCGAGT
CTTAATGAGCACGAGCTTAACGAGCAGCTCGTTCATCCACCCCTCCATAGCAACATAAATATCCACTGGGGTATGTATGACCTCATAATACCGTATG
ATATGAGGATGCATAAATAATCTTAGTATACTGGTTTTTCTTTTTGCTGTAAAATAGCAAAAAAAAACTGAGATTTTACAAAGTACAAAACAGCAAA
AGTGGGAAAAGTACGGGTACATGTTGATCAGGGAAGTCACAAACAGGAACTTCTAACATAACAAATTCAAGAAACTATGGATGGACAATTCTCATAC
TTAAACTAGATATCGACAACAGCCAAATGAAAACTTGCAAGTTGATTGCTAAAGCATCACAGGATTATAACAGAAATAAGGATGAAGTGTTTGACAG
TTTCTACTTTCTACCTTACAGCAGAAACACGCAGATGGCCCTCTGATGAGCTTTTCTCAACGGGCGCAAATCTATGTACACTGTAGGTACTCCAGAA
ACAGAAACAAGCTTTCTGAACAGAATGAGCGATGACCAACCTTTCTCTTCCATCTCCATGCTCTTGATCTTACGGCGATTGAGGATCTTGATTGCGA
CCTTGTGGCCCGTAATTATATGCTCGGCGATCTTCACCTTACCAAAGGACCCGATGCCGAGGGTTTTGCCTATCCGGTAGCCGGCCAGCGGGTTGGC
ATCTCTGCCTGCTGCGTCCAT
>*Traes*CS1B02G364800 protein sequence
MDAAGRDANPLAGYRIGKTLGIGSFGKVKIAEHIITGHKVAIKILNRRKIKSMEMEEKVKREIKILRLFMHPHIIRLYEVIDTPADIYVVMEYVKSG
ELFDYIVEKGRLQEEEARRFFQQIISGVEYCHRNMVVHRDLKPENLLLDSKCNVKIADFGLSNVMRDGHFLKTSCGSPNYAAPEVISGKLYAGPEVD
VWSCGVILYALLCGTLPFDDENIPNLFKKIKGGIYTLPSHLSPLARDLIPRMLVVDPMKRITIREIREHSWFKARLPRYLAVPPPDTAQQVKKLDDE
TLNDVIKMGFDKNQLTESLQKRLQNEATVAYYLLLDNKLRTTSGYLGAEYQESMDSSFSQVSPETPSSASEARQYGSPGFGLRHHFAAERKWALGLQ
SRAHPREIITEVLKALQELNVCWKKIGHYNMKCRWSPGFVETMMHNNNGFGVESAIIEADGLSDKSTHIVKFEIQLYKTRDEKYLLDLQRVSGPQLL
FLDLCSAFLTQLRVL

Supplementary Figure 13. The DNA and deduced protein sequence of *TraesCS1B02G364800* (*TaK1*).

>*Traes*CS4D02G196100 gDNA sequence(340,548,661:340,552,955 bp on chromosome 4D)
ATGGAAGGGAACACTAGAGGAGGCGGGCATTCTGAAGCGTTAAAGAACTACAATGTGGGCAGAACATTAGGTATCGGCACATTTGGAAAAGTGAGGA
TTGCAGAGCATAAGCATACAGGGCACAAAGTTGCTATAAAGATTCTGAACCGTCGTCAAATGAGAACTATGGAAATGGAAGAGAAAGGTATGCCTCC
TTGTACATTAGTCTAGGATACATTCGGCTTTGCTTGCTCAAGATTTCTAGTTATTGGCTTTCAGTGTTGGTCAGATTTGTGAGTGTGTTCCTCATTC
CCAGTTAATCAGTTAGTCGAGATAAAGTACATTAGTCTAGGATACATCCGGCTTCCGTAAAGAAGAAAGTTGACCTTGTGTTGTTGCTGTGAATTAG
TAAAATTTGCAGCTTGAATTTCTTCACAGGCTGAATGTCTAATGCTTTACTTCTATTGTTTTCTCATATCTTGTTTCCAGCAAAGAGAGAGATCAAG
ATATTGAGGTTGTTCATTCACCCTCATATCATCCGGCTTTATGAGGTCATTTACACACCTACAGATATATTTGTTGTGATGGAATATTGCAAGTATG
GTGAGCTATTCGACTGCATTGTTGAGAAAGGGCGGTTACAGGAAGATGAGGCTCGTCGAATCTTCCAGCAGGTGCCTTTTTCTGCTCTTGGCATGTA
GGAACACATTTTTTGGGCATAACAATACGAACTTTGCTGAGCTTCTTATTTTGATCGTGAAATGTATGGATATGCTGGGCAATTGTCCAGTGTCCAC
ATTCCTCACGTGCTGACTCTGCAAATCCTGATCAATTTGCTCAGTTTGCATTCATCTAATCTTTAGCTCATGACCTGTACTACATTGAGTTGAAAGC
TTTCCAAGGTGATTGCTTTGTCGTTTGGGTTGAACTGATCAAAAAGTTGACTTCTTCTTGATGCGTCTATTGTTTTAGTATCACTCTGGTGTCTTGT
TCATTTGATGCCTTGGTTATCTTAGTATCACTAGTTAAATCATTTTTTGTTTAAATGGCATCTTCTTCTCTATGCCTTGATTGTCTTAGTATCGGTA
CTTAAATCAAATTGTGCTTTAAAAATATAAAGAGATGGTATATCCTTCTGTAGGCGAATATGTAATTTGAAGGATTCTTTCTGATCACTGGATGCAT
ATTCAGGTTTTGTCTCAATACTTTTTTTATTTAAATTTTCTTGCACAGATTATATCTGGTGTTGAATACTGCCACAGAAACATGGTTGCTCATCGTG
ATCTAAAGCCAGAAAACCTGTTACTTGATTCCAAATATAATGTGAAACTTGCCGACTTTGGGTTAAGTAATGTCATGCATGATGGCCATTTTCTGAA
GACTAGCTGCGGGAGTCCAAACTATGCTGCACCAGAGGTATTCACTAATTTGTCTTACTTGAGTCTTTATCCTTGTGCTTCCTGACACGCAATGTTT
GCGTTAATAGCTTAGTGAGTGTTTCTGTGCAGGTTATCTCAGGTAAATTATATGCTGGACCTGAGGTTGATGTTTGGAGCTGTGGGGTGATACTTTA
TGCTCTTCTTTGTGGCACTCTTCCATTTGATGATGACAATATTCCCAAACTGTTCAAAAAGATAAAGGTGAGACAGAACACATGTAGATTAAGTTGG
ACCTGACAAGTGTATACCTTTTGTTCTGAAAATAAAAAAACGTTCATGTAGATTAGTTGGACTTTAGCATTCATTTTTTATCTGTAGATTTGACGCC
CCCTTGTTTGGTGTTCCAGGGAGGCATCTATATCCTTCCAAGTCATTTATCTGCTCTTGCAAGGGATTTGATCCCAAGAATGCTTGTTGTTGATCCT
ATGAAGAGAATCACAATTCGTGAAATTCGAGAACACCCATGGTTTCAGAATCGCCTTCCTCGCTACCTGGCAGTGCCTCCACCAGACACGGCGCAGC
AAGCCAAAATGGTACCCATCTATTTCAGATTTCAAGTGACTATCTTGTGTGCACAGTAAAACTTCTCAACCTTTATCGACTAATATATACAGGATCT
TGGATGGATCATAGGAATAACATCGATACTTTTGTCATGAAATGTTATCTTTTATGGGCATAATCTTCATATAATATTTGCAAACACAATATTATAG
AAATATATGGTCAAGTTGTGGCTCAAAGATTCTTTTTTCTCAAACATGCACCAAAGTGCATGGTCATTTCGTATTAAGAAGAGAACGCCGAGAGTCT
GAGTAGGCAACTTTTGAAGTGGATGTCCACCTTCATGAACGTGGTTAAAGCGGGAAGTTTTCCTCCCTTGACGGTACTTTGTTAGGTAGAAATGAGG
CATCATGCAGAGGCTGCCCCTCGAACTTGAGCTCACGGGCCTGTGCTCTAGCCAACCGAGCACTGCTCGGTTCTCTAAAACAAGATCAGTTAGGACT
AGCCCTTGGTGGTATTTTTAACAGAAGAAACAACCTTGACACCCTGTTGAGTCGAGATTCGGACGCGGGTGGGCTGGCTGCATACCGCATGCCTTGC
CAACAGAGCGATGCTCCGTTCTCAGATATCCACCTTCATAGCTCTTCTAACCTTTTATTTGCGAGTAGTCCACATGCCGTCCTCTTGGCCATGTACA
TAATTGTAAAAAAAAACTATACTGCTTAAGTGAAAGGCACTGCTCCTGCTGGTTACTAGGGAAAAAACTAGCCAAAAAACAAAACTATCCAGCAGCT
GTGGCTCAAAGATTTATGAGAAGGGGAAAGATTATGAAAGTTAAACATCATGTAATAATATGAAGTGAAGGGAGTAATTCCTTACTACTAGTAATCT
ATCCCAACGAGATGAAAAGGTAGCACCTAGTACGTTAAGTCTCCTTGCAATGCCGCTTTTATTTATCAAAAATTTCTTTTACTAGCAAGCTTTAAAA
TGCTATTTCTATGTCAATGTATATGCTGATTTGCTCTATGTTCATTTCTGAAGATTGATGAAGATACACTTAAAGAGATTGTCAACCTGGGATATGA
TAAAGCCCATGTGTGTGAATCATTGTGCAATAGGCTGCAAAATGAGGTATATGCAAACAAAAGTCAGTGCTTCATTCCATTCAGTTATTTCATAATT
GACTTTAGACAATGTTTCCAGGCAACTGTTGCATATTACTTACTCTTGGACAATCGGTTCCGGGCCACTAGTGGCTATTTGGGGGCTGACTATCTAC
AATCAATGGTTAGTTCACATTTGCATCATCTGTGACTTGTTTAATTCATAAGGTTTAAGCACTATTAACTTTCTGAATGGATGGTTCTCCAGGGTAG
GAGTTTTAATCAGTTTACTTCATCGGAATCAGCAAGCCCAAGTACCAGGCAGTATCTTCCAGCAAGCAATGATTCTCAAGGCAGTGGCTTGCGGCCA
TATTACCCCGTTGAAAGAAAATGGGCTCTTGGGCTCCAGGTCTTGCACATTATCCACTTTACTTTGCTTTGTTTTGCACATTCAGCTGTTGAGCTAA
CGCATCACTTATCCACCAGTCTCGAGCTCAACCTCGTGAGATAATGATCGAGGTTCTAAAGGCACTTCAAGAATTAAATGTCTGCTGGAAGAAGAAT
GGACACTACAACATGAAATGCAGGTGGTGCCCTGGGTTTCCTCAGGTCAGTGATATGTTAGATGCCAACCACAGCTTTGTTGATGACTCTACCATCA
TGGATAACGGCGATGCTAATGGGAGGCTACCTGCCGTGATCAAGTTTGAAATCCAGGTGTGCAATCTCACCTTCTAATACTACTGTATTCTTTTTGC
AATAACAGAAATATGGATTTTCTACTCAATGTCACCTTCGCAGTCGTATCACTAGATAAAATAACGTTTTTGCTGAAGCAAAGGCTGGCCAATATAT
TCTTATTCGCTTCGTAATTGTTACAAAGTATTGTCTGCATGTGTCTGCTTTCTAACCAGTATATCCTGCATCTGCATGTGTCTGCTGTTGCCAACAA
GTATTGTCTGCTGTTGCCATATTTGATAAAACGAACTTCCCAAGTTTTAGTGCTGCACAGTATTGGTTGGTCATACATCTATCGTTAACCTAGTGTG
ATATTATTCGTTAATGCCTGTATACTTCAGATGTTAACAGTTCGCCGGCATGGTGCTGTATATGCTCTCTATTTGCCTCTGAATTTATGTATTTGTT
TTTTCTTCAGCTTTACAAGACCAAGGATGACAAGTACCTGCTAGATATGCAGAGAGTTACTGGACCTCAGCTCCTCTTTCTGGATTTTTGCGCGGCC
TTCCTTACCAACCTTAGGGTTCTATAG
>TraesCS4D02G196100 protein sequence
MEGNTRGGGHSEALKNYNVGRTLGIGTFGKVRIAEHKHTGHKVAIKILNRRQMRTMEMEEKAKREIKILRLFIHPHIIRLYEVIYTPTDIFVVMEYC
KYGELFDCIVEKGRLQEDEARRIFQQIISGVEYCHRNMVAHRDLKPENLLLDSKYNVKLADFGLSNVMHDGHFLKTSCGSPNYAAPEVISGKLYAGP
EVDVWSCGVILYALLCGTLPFDDDNIPKLFKKIKGGIYILPSHLSALARDLIPRMLVVDPMKRITIREIREHPWFQNRLPRYLAVPPPDTAQQAKMI
DEDTLKEIVNLGYDKAHVCESLCNRLQNEATVAYYLLLDNRFRATSGYLGADYLQSMGRSFNQFTSSESASPSTRQYLPASNDSQGSGLRPYYPVER
KWALGLQSRAQPREIMIEVLKALQELNVCWKKNGHYNMKCRWCPGFPQVSDMLDANHSFVDDSTIMDNGDANGRLPAVIKFEIQLYKTKDDKYLLDM
QRVTGPQLLFLDFCAAFLTNLRVL

Supplementary Figure 14. The DNA and deduced protein sequence of *Traes*CS4D02G196100 (*TaK4*).

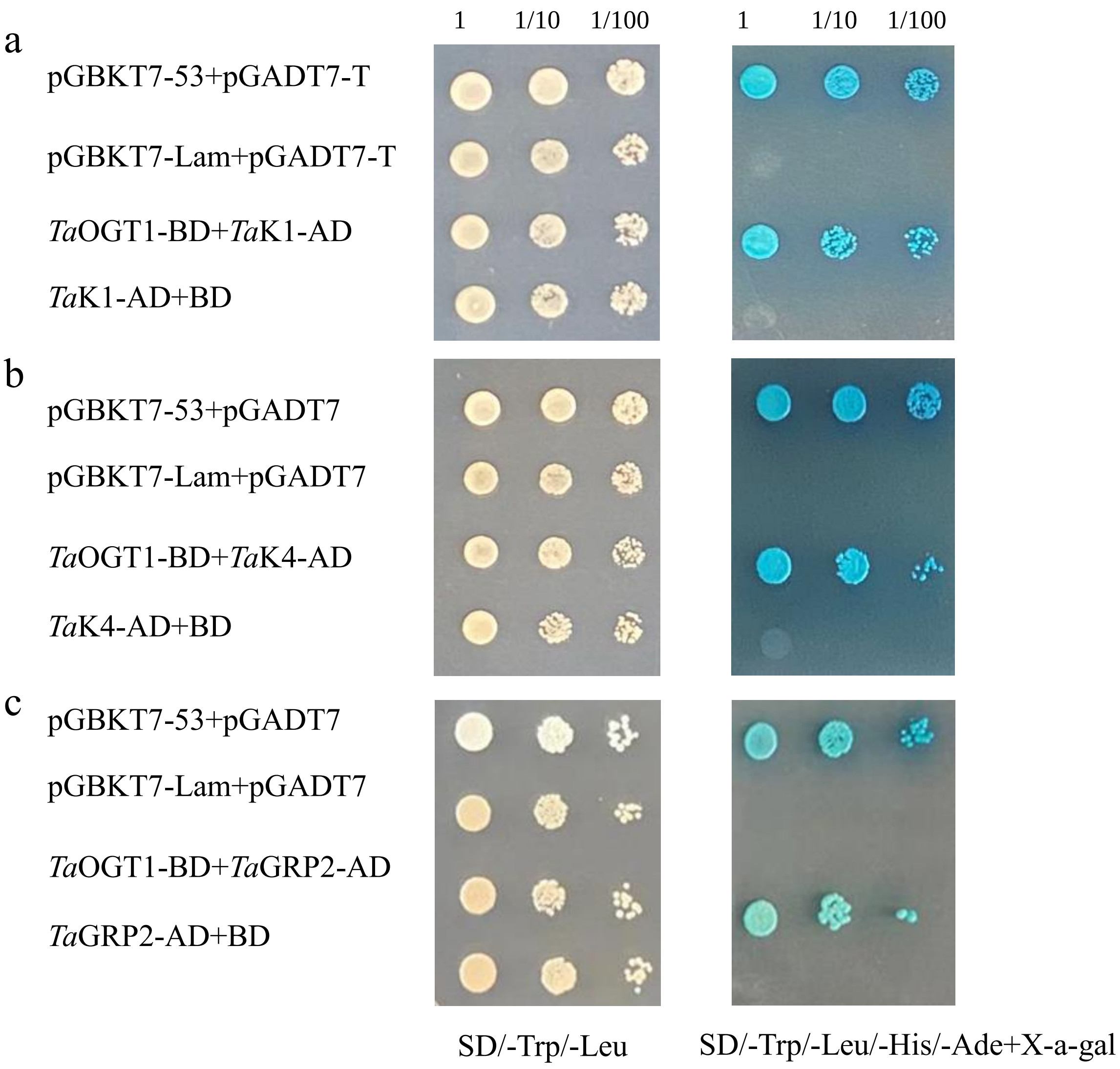

Supplementary Figure 15. Interactions of *Ta*OGT1b with *Ta*K1, *Ta*K4, and *Ta*VRT2. a. *Ta*K1. b. *Ta*K4. c. *Ta*VRT2. Co-transformed cells were grown on plates lacking two amino acids (–Leu/–Trp), and plates lacking four amino acids (–Leu/–Trp/–His/–Ade). Four colony solutions diluted with different folds (1, 1/10, 1/100) were inoculated on the same plate for each of the protein pairs. *Ta*OGT1 protein expressed in the BD vector was tested to interact with *Ta*K1 (a), *Ta*K4 (b) or *Ta*VRT2 (c) in the AD vector, and the empty BD vector was used as a negative control. pGBKT7-53+pGADT7-T was used as a positive control to show interacting proteins, and pGBKT7-Lam+pGADT7-T was used as a negative control to show on interaction between proteins.

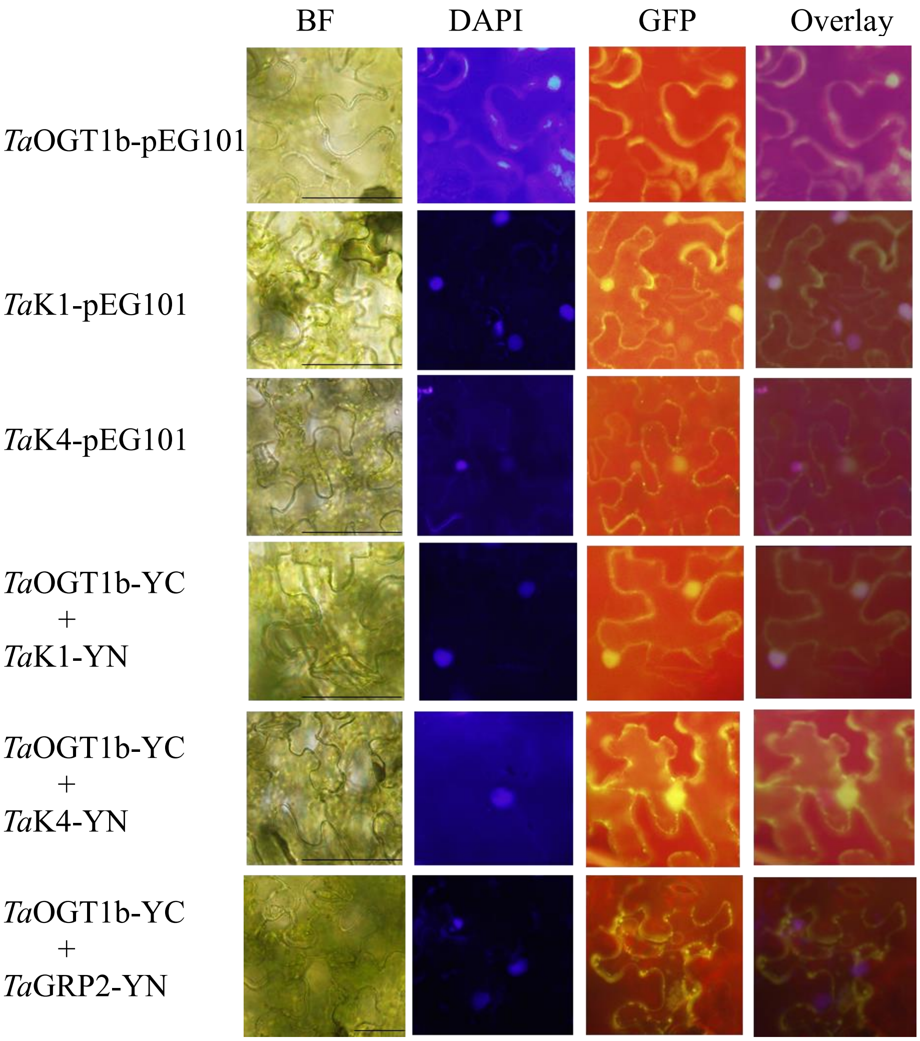

Supplementary Figure 16. The subcellular localization and interactions of proteins. *Ta*OGT1b, *Ta*K1, and *Ta*K4 each was fused with a yellow fluorescent protein YFP in pEG101 vector to express in tobacco leaves for the subcellular localization. The yellow fluorescent signals indicate locations of the expressed proteins. The BiFC interaction of *Ta*OGT1b fused with YC was tested for interaction with *Ta*K1, *Ta*K4 or *Ta*GRP2 fused with YN. YC, the C-terminal end fragment of YFP, and YN, the N-terminal fragment of YFP. When *Ta*OGT1b-YC and *Ta*K1-YN, *Ta*K4-YN or *Ta*GRP2-YN were simultaneously expressed in the same cell, the fluorescent signals were observed, indicating that the proteins had interactions. The leaves were infiltrated with *A. tumefaciens* carrying constructs, and were imaged using a fluorescent microscope fitted with bright field (BF), YFP, and DAPI filters to indicate the locations of the cell, YFP, and nucleus stained with 4′, 6-diamidino-2-phenylindole (DAPI), respectively. The overlay images were used to align the locations of YFP with the DAPI-stained nucleus. Scale bars in all images are 50 μm. This experiment was repeated three times and observed similar results.

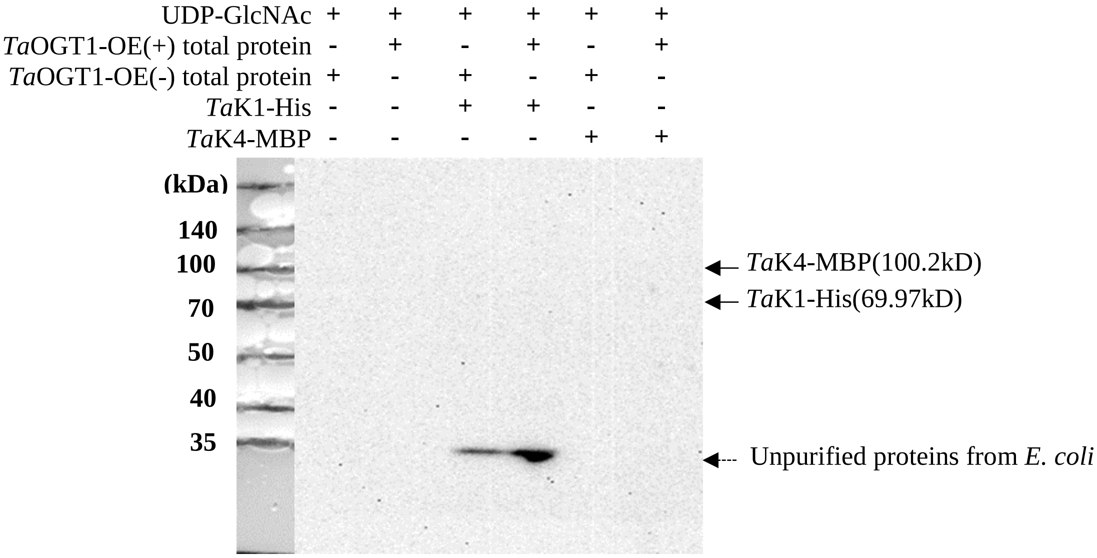

Supplementary Figure 17. GlcNAcylation of *Ta*K1 and *Ta*K4 by *Ta*OGT1. *Ta*OGT1 was the total protein from leaves of the transgenic wheat plant *Ta*OGT1-OE(+), or the non-transgenic wheat plant *Ta*OGT1-OE(-). TaK1-His was used as a regulator. The location of *Ta*K1 and *Ta*K4 on the blot is indicated with an arrow. The GlcNAcylated proteins were detected with the antibody against *O*-GlcNAc CTD110.6. No GlcNAcylation signal was observed for *Ta*K1 or *Ta*K4, but the GlcNAcylation signal was observed for a protein that was from the *Ta*K1 samples extracted from *E. coli*. The unexpected result indicates that *Ta*OGT1 was able to GlcNAcylate proteins in *E. coli*. Full blot image is provided as a Source Data file.

>*Traes*CS4B02G020300 (*TaGRP2*) gDNA sequence (chromosome 4B)

ATGGCGGACGTCGAGTACCGCTGCTTCGTCGGCGGCCTCGCCTGGGCCACCGACGACCAGTCCCTCCAGAACGCCTTCTCCAAGTACGGCGACGTCA
TCGACTCCAAGGTACCGTCCCCTCCCCGATCCGCCCGATAGATCTGTAGATTCGAGATGGATCTGTGGTGTTGCGCTTGTGGAACCCTAGATCTGAC
GGTTTTGTTGGTGCAGATCATCACTGACAGGGAGACGGGCCGATCCCGCGGGTTCGGGTTCGTCACCTTCGCGTCGGACGAGGCGATGCGCCAGGCG
ATCGAGGCCATGAACGGCCAGGACCTGGACGGCCGCAACATCACCGTCAACGAGGCGCAGTCCCGTCGCTCCGGCGGAGGAGGCGGCGGCGGCTTCG
GCGGCGGCGGTGGCGGCTACGGGGGCCAGCGCCGTGAGGGCGGCGGCGGCGGCGGCTACGGCGGCGGCGGCGGCGGCTACGGAGGTGGCCGCAGCGG
CGGCGGCGGCGGCTACGGCAGCCGTGACGGCGGCGGCTACGGAGGCGGCGGCGGCGGCGGCTACGGCGGCAGCCGCGGCGGCTCCGGCGGCGGCAAC
TGGAGGGAGTGA

>*Ta*GRP2-protein, Glycine-rich RNA-binding protein

MADVEYRCFVGGLAWATDDQSLQNAFSKYGDVIDSKIITDRETGRSRGFGFVTFASDEAMRQAIEAMNGQDLDGRNITVNEAQSRRSGGGGGGGFGG
GGGGYGGQRREGGGGGGYGGGGGGYGGGRSGGGGGYGSRDGGGYGGGGGGGYGGSRGGSGGGNWRE

Supplementary Figure 18. *TaGRP2* gene and protein in wheat (cultivar: Billings)

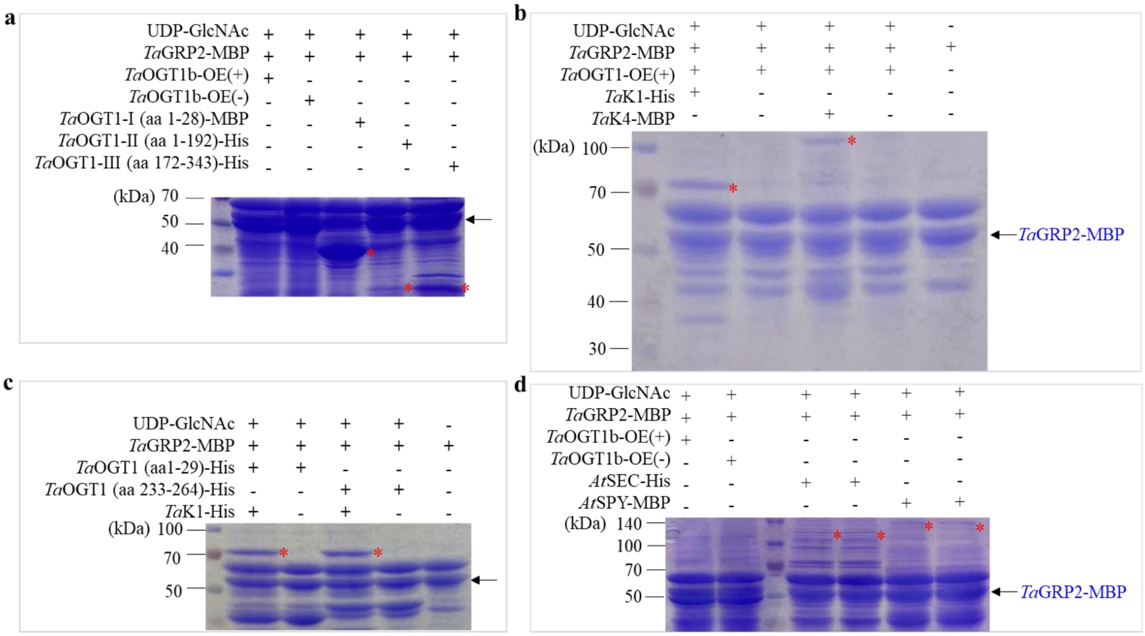

Supplementary Figure 19. The images of Coomassie Brilliant Blue stained protein gels. Coomassie Brilliant Blue (CBB) R-250 (Bio-Rad) was used as a dye to stain with SDS-PAGE gels before tested with antibodies shown in Fig. 5. Presence and absence of the particular protein in the reaction is indicated by '+' and '-', respectively. a. Comparison of OGT enzyme activity among different *Ta*OGT1 proteins. *Ta*OGT1 proteins from a transgenic plant (+), proteins from a non-transgenic plant (-), and three different *Ta*OGT1 protein fragments labeled with red star, were expressed in *E. coli*: aa 1–28, aa 1–192, and aa 172–343. b. Effects of *Ta*K1 and *Ta*K4 on GlcNAcylation by *Ta*OGT1. *Ta*OGT1 was extracted from total protein from leaves of transgenic wheat plants, and *Ta*K1 or *Ta*K4 proteins labeled with red star were added to the GlcNAcylation reaction. c. Comparison of OGT enzyme activity of two protein fragments of aa 1–28 and aa 233–264. *Ta*K1 proteins labeled with red star were added to the GlcNAcylation reaction. d. Comparison of *At*SEC and *At*SPY proteins expressed in *E. coli* with *Ta*OGT1 proteins from a transgenic plant (+), a non-transgenic plant (–). *At*SEC and *At*SPY proteins labelled with red star were added to the GlcNAcylation reaction. *Ta*GRP2 protein was used as a substrate in each GlcNAcylation reactions. Full gel images are provided as a Source Data file.

>*Ta*OGT1b protein sequence
MRFTAGGSSTRSWQPKPTADTTDLRFWLRWRVAVCALWVLCCVAAAAYLVWRHEGPRAHRRPGAAKQDAAAQGRRRPDGLLYDDEAWRPCLRDIHPAWLLAYRLVSFFVLF
SLLIVIVISDGGNIFYYYTQWTFILVTVYFGLATTLSIYGCSKFAACNAVAAMSDAEQGPYAVHGAAPKPVVDREDDGTREVAGFWGYLLQIIYQTNAGAVMLTDCVFWFI
IFPFLTIKDYSMNFLLIGMHSVNAVFLLGEASLNSLRFPWFRIAYFFLYTALYVVFQWIVHASTPTWWPYPFLDLSSNLAPLWYFAVAFMQLPCYLIFRLVMNLKHHLLAK
HFPDSMVLGY
>*Ta*OGT-P1 (aa. 1-28)
MRFTAGGSSTRSWQPKPTADTTDLRFWL
>*Ta*OGT-P2 (aa. 1-192)
MRFTAGGSSTRSWQPKPTADTTDLRFWLRWRVAVCALWVLCCVAAAAYLVWRHEGPRAHRRPGAAKQDAAAQGRRRPDGLLYDDEAWRPCLRDIHPAWLLAYRLVSFFVLF
SLLIVIVISDGGNIFYYYTQWTFILVTVYFGLATTLSIYGCSKFAACNAVAAMSDAEQGPYAVHGAAPKPVVDREDDGTRE
>*Ta*OGT-P3 (aa. 172-343)
*M*YAVHGAAPKPVVDREDDGTREVAGFWGYLLQIIYQTNAGAVMLTDCVFWFIIFPFLTIKDYSMNFLLIGMHSVNAVFLLGEASLNSLRFPWFRIAYFFLYTALYVVFQWI
VHASTPTWWPYPFLDLSSNLAPLWYFAVAFMQLPCYLIFRLVMNLKHHLLAKHFPDSMVLGY
>*Ta*OGT-P2 (aa. 234-264)
MNFLLIGMHSVNAVFLLGEASLNSLRFPWFR

Supplementary Figure 20. Two active sites of *Ta*OGT1 protein. Sequences of five protein fragments were tested for GlcNAcylation enzyme activity. One active site is highlighted in yellow and the other active site is highlighted in green.

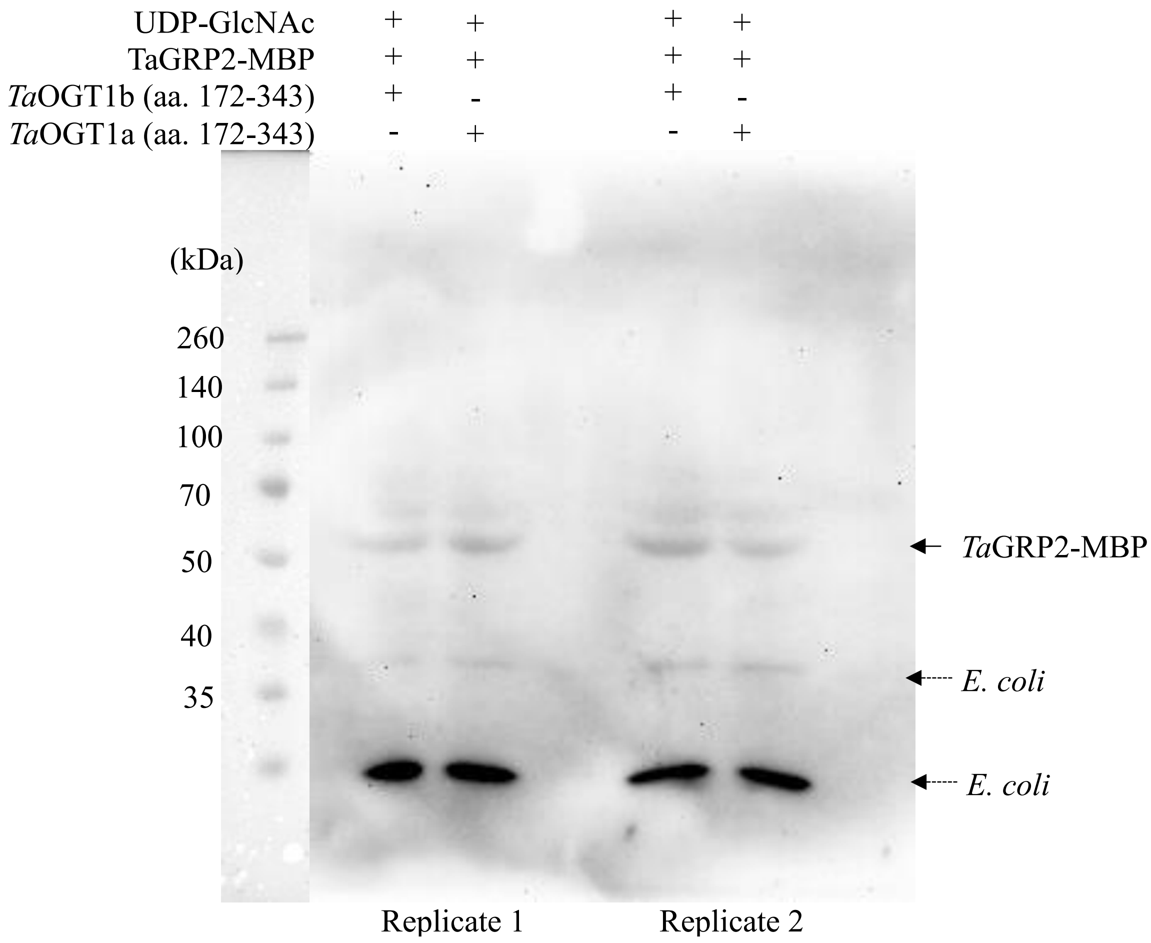

Supplementary Figure 21. Comparison of GlcNAcylation enzyme activity between *Ta*OGT1a and *Ta*OGT1b. *Ta*GRP2 protein was expressed using MBP vector. The purified *Ta*GRP2 protein was used as a substrate in each GlcNAcylation reaction. *Ta*OGT1a and *Ta*OGT1b protein fragments were expressed in *E. coli* and then purified. The GlcNAcylated proteins were detected with the antibody against O-GlcNAc CTD110.6. Presence and absence of the particular protein in the reaction is indicated by '+' and '-', respectively. The locations of GlcNAcylated proteins on the blot are indicated with an arrow. In addition to *Ta*GRP2, proteins from *E. coli* were GlcNAcylated by *Ta*OGT1a and *Ta*OGT1b. No significant difference in GlcNAcylation enzyme activity was observed between *Ta*OGT1a and *Ta*OGT1b.

Supplementary Table 1. *P* value from a *t*-test to determine significance level for comparing mean difference between treatments.

Gene	Comparison	Condition	1 wk	3 wk	6 wk
TaOGT1	N vs. P	GH	2.1E-07	7.3E-04	3.2E-07
		CR	6.8E-09	5.1E-06	3.5E-05
	GH vs. CR	N	0.17	0.05	0.982
		P	0.396	0.001	0.269
Glucose	N vs. P	GH	0.131	0.001	0.001
		CR	0.021	0.001	7E-08
	GH vs. CR	N	0.1	0.498	0.081
		P	0.042	0.081	0.053
Sucrose	N vs. P	GH	0.002	0.001	4.8E-07
		CR	0.004	2.9E-06	5.7E-05
	GH vs. CR	N	0.141	0.0019	3.2E-05
		P	0.029	3.4E-06	1.7E-06
Fructose	N vs. P	GH	0.119	0.298	0.624
		CR	0.008	0.091	0.277
	GH vs. CR	N	0.002	0.127	0.005
		P	0.027	0.911	0.001
vrn1	N vs. P	GH	0.001	0.023	0.001
		CR	4.7E-05	0.001	0.019
	GH vs. CR	N	1.1E-06	0.009	0.006
		P	8.8E-06	0.011	0.084
vrn3	N vs. P	GH	0.001	0.001	0.004
		CR	0.002	5.9E-05	0.001
	GH vs. CR	N	8.5E-05	0.004	0.002
		P	2.6E-05	0.001	0.005
VRN2	N vs. P	GH	0.018	0.214	0.124
		CR	0.098	0.769	0.467
	GH vs. CR	N	0.001	8.5E-06	0.001
		P	0.001	0.00`	0.001
PPD1	N vs. P	GH	0.001	0.012	0.001
		CR	0.07	0.067	0.007
	GH vs. CR	N	0.201	0.009	0.266
		P	0.751	0.692	0.31
TaVRT2	N vs. P	GH	0.643	0.209	0.033
		CR	0.672	0.824	0.448
	GH vs. CR	N	0.105	2.7E-05	9.5E-06
		P	0.028	1E-05	0.001
TaGRP2	N vs. P	GH	0.001	0.004	5.9E-05
		CR	0.001	0.032	0.009
	GH vs. CR	N	0.864	0.929	0.026
		P	0.522	0.962	0.452

The two tailed unpaired Students' *t*-test was performed for each pair of treatments, which data are from mean of three biological replicates and two or three technical replicates (*n*=6-9). N represents non-transgenic plant, P represents transgenic plants, GH represents greenhouse for plants that were not vernalized, and represents cold room for plants that were vernalized. *P* value equal to or smaller than 0.05 for significant difference is indicated in red, and *P* value equal to or smaller than 0.01 for highly significant difference is indicated in blue.

Supplementary Table 2. Pearson correlation coefficient to measure linear correlation between two variables.

	UDP	*TaOGT1*	Glucose	Sucrose	Fructose	*vrn1*	*VRN2*	*vrn3*	*PPD1*	*TaVRT2*	*TaGRP2*
UDP	1	0.393	-0.113	-0.405	0.115	0.975	-0.215	0.911	0.609	-0.406	0.071
TaOGT1		1	-0.355	-0.557	-0.033	0.311	0.003	0.647	0.901	-0.213	0.871
Glucose			1	0.677	0.599	-0.155	0.103	-0.217	-0.452	-0.055	-0.595
Sucrose				1	0.561	-0.375	0.441	-0.537	-0.74	0.452	-0.569
Fructose					1	0.162	0.086	0.068	-0.097	0.119	-0.35
vrn1						1	-0.194	0.843	0.562	-0.352	0.005
VRN2							1	-0.247	-0.082	0.723	0.186
vrn3								1	0.751	-0.5	0.326
PPD1									1	-0.274	0.764
TaVRT2										1	0.058
TaGRP2											1

The mean value of each variable was from three biological replicates and two technical replicates of transgenic and non-transgenic plants with and without vernalization (n = 12). *P* value that reaches a significant level for positive linear correlation is indicated in blue. *P* value that reaches a significant level for negative linear correlation is indicated in red.

Supplementary Table 3. *P* value to determine significance level for linear correlation between two variables.

	UDP	*TaOGT1*	Glucose	Sucrose	Fructose	*vrn1*	*VRN2*	*vrn3*	*PPD1*	*TaVRT2*	*TaGRP2*
UDP	1	n.s.	n.s.	n.s.	n.s.	p<0.00001	n.s.	p<0.0001	p=0.036	n.s.	n.s.
TaOGT1		1	n.s.	n.s.	n.s.	n.s.	n.s.	p=0.023	p<0.0001	n.s.	p=0.0002
Glucose			1	p=0.015	n.s.	n.s.	n.s.	n.s.	n.s.	n.s.	n.s.
Sucrose				1	n.s.	n.s.	n.s.	n.s.	p=0.006	n.s.	n.s.
Fructose					1	n.s.	n.s.	n.s.	n.s.	n.s.	n.s.
vrn1						1	n.s.	p=0.0006	n.s.	n.s.	n.s.
VRN2							1	n.s.	n.s.	p=0.0007	n.s.
vrn3								1	p=0.038	n.s.	n.s.
PPD1									1	n.s.	p=0.004
TaVRT2										1	n.s.
TaGRP2											1

P value that reaches a significant level or a highly significant level for linear correlation is provided in the form. The n.s. indicates a non-significant level.

Supplementary Table 4. Primers used for markers.

Markers	Primer	Primer Sequence (5'-3')	Product size (bp)
XDH-KSAP2	XDH-6A-DF2	GAAGGTGACCAAGTTCATGCTATGGAGTTTGTGAGGGGGAT	153
	XDH-6A-BF2	GAAGGTCGGAGTCAACGGATTATGGAGTTTGTGAGGGGGAG	
	XDH-6A-R2	CTAATTTTGTGGAGGCTCGCG	
6A-KASP13	HD-DF13	GAAGGTGACCAAGTTCATGCTGTGGCAGCAAGCTTTTCACA	156
	HD-BF13	GAAGGTCGGAGTCAACGGATTGTGGCAGCAAGCTTTTCACG	
	HD-R13	AGACGACGAAATTGCTGGAGT	
6A-KASP4	HD-6A-DF4	GAAGGTGACCAAGTTCATGCTCGAATTGGCAGGAAGAATAACAACT	99
	HD-6A-BF4	GAAGGTCGGAGTCAACGGATTCGAATTGGCAGGAAGAATAACAACC	
	HD-6A-R4	TGTAGGAGACGCGTGCGC	
6A-KASP3	HD-6A-DF3	GAAGGTGACCAAGTTCATGCTATGCCTCATCGCAGTTCACA	91
	HD-6A-BF3	GAAGGTCGGAGTCAACGGATTATGCCTCATCGCAGTTCACC	
	HD-6A-R3	GAAGCAGTTCGAGCCACCT	
6A-400M1	6A-400M1-F1	GTCCCAAGTCGGGGACGTCAATATATA	1070
	6A-400M1-R1	GGTGCCGTCGTCGCCAAA	
6A-400M2	6A-400M2-F2	GACGCGCGCCATCGCTTT	926
	6A-400M2-R2	CTTAGCTAGTAGGGCACGGAAGCATT	
6A-SEQ7	6A-SEQ7-F7	TTCAAATTATAACTGATCTAGTTTTGAC	430
	6A-SEQ7-R7	GAAATTAGGACTATTACACAATAATTTAAAC	
TaOGT1	168Ind-F1	CTTCTCATGATTACATCAACTACTGATCTTA	276/108
	168Ind-R1	AGGCAATTTCAAAATATTAAAAATGAAGTAGC	
vrn-A1	VRN-A1F4	CAACTTGTTTGGGACTAAAGGC	375
	VRN-A1R42	CTGCAACTCCTTGAGATTCAAAG	
vrn-D3	VRN-D3-F6	CTTCTATTCACATGTTTCGTTCATG	402
	VRN-D3-R8	ACGAGCACGAAGCGATGGATCGC	
PPD-D1	PPD-D1-F	ACGCCTCCCACTACACTG	414
	PPD-D1-R1	GTTGGTTCAAACAGAGAGC	
	PPD-D1-R2	CACTGGTGGTAGCTGAGATT	

Note: The sequences highlighted in gray are tails of the forward primer and reverse primer HEX.

Supplementary Table 5. Primers used for cloning, protein interactions, EMSA, and gene expression.

Gene/ locus	Primer	Primer Sequence (5'-3')	Product size (bp)
*Ta*OGT1b	attB1-F1	GGGGACAAGTTTGTACAAAAAAGCAGGCTTCATGCGGTTCACGGCG	1032
	attB2-R1	GGGGACCACTTTGTACAAGAAAGCTGGGTCCTAGTATCCTAGGACCATGCTATCC	
GUS	HindIII-F1	CGAAGCTTAGGCACATCTATCCTGTGACC	1327
	KpnI-R1	CGGGTACCAGGCAATTTCAAAATATTAAAAATGAAGTAGC	/1159
LUC	attB1-F1	GGGGACAAGTTTGTACAAAAAAGCAGGCTTCATGGAAGATGCCAAAAACATTAAGAAGG	1653
	attb2-R1	GGGGACCACTTTGTACAAGAAAGCTGGGTCTTACACGGCGATCTTGCC	
*vrn1**	F1	GAATAAAGTTCTCCAGAAGGAACTCGTG	104
	R2	GCATGAAGGAAGAAGATGAAGAGCTG	
*VRN2**	F	CCGACACATGGCTCACCTAGTG	95
	R	TTGCTTCATTGCTAATAGTGTTTGT	
*vrn3**	F	CAGCAGCCCAGGGTTGAG	72
	R	ATCTGGGTCTACCATCACGAGTG	
*PPD1**	F1	CCCAACAGGCAGCTAGAGC	161
	R1	CCTCTGTTCCTCCTGGATCG	
*TaVRT2**	F	CAGAGGAAAATATGCGCTTG	155
	R	ATCATTGTCCTGCGAGCTT	
*TaGRP2**	F	AACGCCTTCTCCAAGTACGG	101
	R	TCCGACGCGAAGGTGAC	
*Actin**	F2	GGAACTGGCATGGTCAAGGCTG	107
	R2	CCCATCCCCACCATCACACC	
*Ta*OGT1	168Ind-F1	CTTCTCATGATTACATCAACTACTGATCTTA	276
	168Ind-R1	AGGCAATTTCAAAATATTAAAAATGAAGTAGC	
*Ta*VRT2	EcoRI-F1	ACACTGAATTCATGGCGCGGGAGAGG	681
	BamHI-R1	CCGGGATCCTTACTTCCAAGGTAACGCTAGTTTCA	
*Ta*OGT1	EcoRI-F1	CGGAATTCATGCGGTTCACGGCGGG	1032
	BamHI-R1	CGGGATCCCTAGTATCCTAGGACCATGCTATCCGGGAA	
*Ta*OGT1(aa. 1-28)	EcoRI-F1	CGGAATTCATGCGGTTCACGGCGGG	84
	BamHI-R2	CGGGATCCCTACAGCCAGAAGCGCAGGTC	
*Ta*OGT1(aa. 1-192)	NdeI-F1	AGCAGCGGCATATGCGGTTCACGGCGGG	576
	BamHI-R3	CGGGATCCCTACTCCCTCGTGCCGTCGT	
*Ta*OGT1(aa. 172-343)	NdeI-F2	AGCAGCGGCATATGTACGCCGTCCACGGGG	519
	BamHI-R1	CGGGATCCCTAGTATCCTAGGACCATGCTATCCGGGAA	
*Ta*OGT1(aa. 233-264)	EcoRI-F5	ACACTGAATTCATGAATTTTCTATTGATAGGAATGCACTCG	93
	BamHI-R5	CCGGGATCCCTACCGGAACCACGGGAAG	
AtSEC	NdeI-F1	GCGGCATATGATCTCGTCCAAAAACGGAGC	2934
	BamHI-R1	CGCGGATCCTTATCTGTCATGTGGGAATTCTAGGTCG	
AtSPY	EcoRI-F1	ACTGAATTCATGGTGGGACTGGAAGATGATACTG	2745
	BamHI-R1	CGCGGATCCCTAGCTAGTGGAGTCCATTCTCTTTGAG	
TaK1	Y2H-NdeI-F1	AGCAGCGGCATATGGACGCAGCAGGCAG	1503
	Y2H-BamHI-R1	CGCGGATCCTCAGAGGACTCTCAGCTGGGTTAGAAAG	
TaK4	Y2H-EcoRI-F1	CGGAATTCATGGAAGGGAACACTAGAGGA	1530
	Y2H-BamHI-R1	CGGGATCCCTATAGAACCCTAAGGTTGGTA	
*Ta*GRP2	Y2H-EcoR1-F1	ACACTGAATTCATGGCGGACGTCGAGT	492
	Y2H-BamH1-R1	CGCGGATCCTCACTCCCTCCAGTTGCC	
*Ta*OGT1	BiFC-F1	GGGGACAAGTTTGTACAAAAAAGCAGGCTTCATGCGGTTCACGGCG	1029
	BiFC-R1	GGGGACCACTTTGTACAAGAAAGCTGGGTCTATCCTAGGACCATGCTATCCG	
*Ta*K1	BiFC-F1	GGGGACAAGTTTGTACAAAAAAGCAGGCTTCATGGACGCAGCAGGCAG	1503
	BiFC-R1	GGGGACCACTTTGTACAAGAAAGCTGGGTCAGGACTCTCAGCTGGGTTAGAAAG	
*Ta*K4	BiFC-F1	GGGGACAAGTTTGTACAAAAAAGCAGGCTTCATGGAAGGGAACACTAGAGGAG	1530
	BiFC-R1	GGGGACCACTTTGTACAAGAAAGCTGGGTCTAGAACCCTAAGGTTGGTAAGGAA	
*Ta*GRP2	BiFC-F1	GGGGACAAGTTTGTACAAAAAAGCAGGCTTCATGGCGGACGTCGAGT	492
	BiFC-R1	GGGGACCACTTTGTACAAGAAAGCTGGGTCCTCCCTCCAGTTGCCG	

Note: Sequences marked in red indicate the restriction enzyme sites for cloning. Sequences highlighted in gray indicate oligonucleotides that are attached to forward and reverse primers used for cloning of the Gateway vector. Sequences of the start codon and stop codon are indicated in red and highlighted in yellow. G091100 indicates the gene *TraesCS6A01G091100*, and G091200 indicates the gene *TraesCS6A01G091200*. * indicates the primers used for gene expression.

Supplementary references

1. Edwards, J.T., Hunger, R.M., Smith, E.L., Horn, G.W., Chen, M.S., Yan, L. 'Duster' Wheat: A durable, dual-purpose cultivar adapted to the Southern Great Plains of the USA. *J. Plant Regist.* **6,** 1-12 (2012).
2. Hunger, R.M., Edwards, J.T., Bowden, R.L., Yan, L., Rayas-Duarte, P., Bai, G., Horn, G.W., Kolmer, J.A., Giles, K.L., Chen, M.S., Jin, Y. 'Billings' wheat combines early maturity, disease resistance, and desirable grain quality for the Southern Great Plains of the USA. *J. Plant Regist.* **8,** 22-31 (2014).
3. Li, G., Wang, Y., Chen, M.S., Edae, E., Poland, J., Akhunov, E., Chao, S., Bai, G.H., Carver, B.F., Yan, L.L. Precisely mapping a major gene conferring resistance to Hessian fly in bread wheat using genotyping-by-sequencing. *BMC Genomics*. **16,** 108 (2015).
4. Fang, T,, Lei, L., Li, G., Powers, C., Hunger,. RM., Carver, B.F., Yan, L. Development and deployment of KASP markers for multiple alleles of Lr34 in wheat. *Theor. Appl. Genet.*133, 2183-2195 (2020).
5. Kan, C-C., Jia, H., Powers, C., Carver, B.F., Yan, L. Genetic characterization and deployment of a major gene for grain yield on chromosome arm 1BS in winter wheat. *Mol. Breed.* **40,** 26 (2020).
6. IWGSC. Shifting the limits in wheat research and breeding using a fully annotated reference genome. *Science* **361,** eaar7191 (2018).
7. Chen, Y.H., Carver, B.F., Wang, S.W., Zhang, F.Q. & Yan, L. Genetic loci associated with stem elongation and winter dormancy release in wheat. *Theor. Appl. Genet.* **118**, 881-889 (2009).
8. Li, G., Yu, M., Fang, T., Cao, S., Carver, B.F., Yan, L. Vernalization requirement duration in winter wheat is controlled by *Ta*VRN-A1 at the protein level. *Plant J.* **76,** 742–753 (2013).
