# Supplementary material for: O-linked N-acetylglucosamine transferase is involved in fine regulation of flowering time in winter wheat
Source: Nat Commun. 2021 Apr 16;12:2303. doi: 10.1038/s41467-021-22564-8 (PMC8052332; doi:10.1038/s41467-021-22564-8)
Supplement: Supplementary file 3 — Reporting Summary [file 41467_2021_22564_MOESM3_ESM.pdf]

nature research

Corresponding author(s): Liuling Yan

Last updated by author(s): Oct 12, 2020

Reporting Summary

Nature Research wishes to improve the reproducibility of the work that we publish. This form provides structure for consistency and transparency in reporting. For further information on Nature Research policies, see our Editorial Policies and the Editorial Policy Checklist.

Statistics

For all statistical analyses, confirm that the following items are present in the figure legend, table legend, main text, or Methods section.

n/a	Confirmed	
☐	☒	The exact sample size (*n*) for each experimental group/condition, given as a discrete number and unit of measurement
☐	☒	A statement on whether measurements were taken from distinct samples or whether the same sample was measured repeatedly
☐	☒	The statistical test(s) used AND whether they are one- or two-sided<br>*Only common tests should be described solely by name; describe more complex techniques in the Methods section.*
☒	☐	A description of all covariates tested
☒	☐	A description of any assumptions or corrections, such as tests of normality and adjustment for multiple comparisons
☐	☒	A full description of the statistical parameters including central tendency (e.g. means) or other basic estimates (e.g. regression coefficient) AND variation (e.g. standard deviation) or associated estimates of uncertainty (e.g. confidence intervals)
☐	☒	For null hypothesis testing, the test statistic (e.g. *F*, *t*, *r*) with confidence intervals, effect sizes, degrees of freedom and *P* value noted<br>*Give P values as exact values whenever suitable.*
☒	☐	For Bayesian analysis, information on the choice of priors and Markov chain Monte Carlo settings
☒	☐	For hierarchical and complex designs, identification of the appropriate level for tests and full reporting of outcomes
☒	☐	Estimates of effect sizes (e.g. Cohen's *d*, Pearson's *r*), indicating how they were calculated

Our web collection on statistics for biologists contains articles on many of the points above.

Software and code

Policy information about availability of computer code

Data collection	*Provide a description of all commercial, open source and custom code used to collect the data in this study, specifying the version used OR state that no software was used.*
Data analysis	*Provide a description of all commercial, open source and custom code used to analyse the data in this study, specifying the version used OR state that no software was used.*

For manuscripts utilizing custom algorithms or software that are central to the research but not yet described in published literature, software must be made available to editors and reviewers. We strongly encourage code deposition in a community repository (e.g. GitHub). See the Nature Research guidelines for submitting code & software for further information.

Data

Policy information about availability of data

All manuscripts must include a data availability statement. This statement should provide the following information, where applicable:

- Accession codes, unique identifiers, or web links for publicly available datasets
- A list of figures that have associated raw data
- A description of any restrictions on data availability

All relevant data and materials presented within the article and its supporting Information files are available on request.

Field-specific reporting

Please select the one below that is the best fit for your research. If you are not sure, read the appropriate sections before making your selection.

☒ Life sciences ☐ Behavioural & social sciences ☐ Ecological, evolutionary & environmental sciences

For a reference copy of the document with all sections, see nature.com/documents/nr-reporting-summary-flat.pdf

Life sciences study design

All studies must disclose on these points even when the disclosure is negative.

Sample size	Duster and Billings were used to generate a population of 192 F2 plants, and derived 273 F3 plants and 153 F4 plants were used for screening of crossovers at the QHd.osu-6A locus. Sixty plants of each critical DH line and 25 plants of each F3:4 recombinant lines were tested under the same greenhouse conditions. Transgenic T1 families were tested under the same conditions and the requirement of statistic analyses.
Data exclusions	No data was excluded.
Replication	Genetic effects of the TaOGT1 on flowering time in the natural populations were tested in the same greenhouse with the same conditions, and all the attempts at replications were successful. Functions of the cloned gene in transgenic wheat plants were confirmed in two individual transgenic events.
Randomization	Each plant in a recombinant line or a transgenic family was tested in one pot or culm that was placed on greenhouse benches in randomized design. Plants in the same line or family were placed as a group for comparison of alleles in different genetic background.
Blinding	The investigators were blinded to group allocations or alleles during collection for phenotypes.

Reporting for specific materials, systems and methods

We require information from authors about some types of materials, experimental systems and methods used in many studies. Here, indicate whether each material, system or method listed is relevant to your study. If you are not sure if a list item applies to your research, read the appropriate section before selecting a response.

Materials & experimental systems

n/a	Involved in the study	
☐	☒	Antibodies
☒	☐	Eukaryotic cell lines
☒	☐	Palaeontology and archaeology
☒	☐	Animals and other organisms
☒	☐	Human research participants
☒	☐	Clinical data
☒	☐	Dual use research of concern

Methods

n/a	Involved in the study	
☒	☐	ChIP-seq
☒	☐	Flow cytometry
☒	☐	MRI-based neuroimaging

Antibodies

Antibodies used	An antibody against O-GlcNAc (CTD110.6 mouse mAb HRP conjugate, Cell Signaling Technology, Danvers, MA) was used to detect O-GlcNAc modification of proteins, and an antibody to Actin (Abclonal, Woburn, MA) was used as a control.
Validation	These antibodies are commonly used in protein research.
